# Supplementary material for: Mining cancer genomes for change-of-metabolic-function mutations
Source: Commun Biol. 2023 Nov 10;6:1143. doi: 10.1038/s42003-023-05475-w (PMC10638295; doi:10.1038/s42003-023-05475-w)
Supplement: Supplementary file 2 — Supplementary Material [file 42003_2023_5475_MOESM2_ESM.pdf]

Supplementary Material

Mining Cancer Genomes for Change-of-Metabolic-Function Mutations

Kevin J. Tu, Bill H. Diplas, Joshua A. Regal, Matthew S. Waitkus, Christopher J. Pirozzi, Zachary J. Reitman

Table of Contents **Page**

Table of Contents	Page
Supplementary Figure 1. Graphical abstract of the METIS pipeline and results	2
Supplementary Figure 2. Metabolomic changes in CBL cells	3
Supplementary Figure 3. Metabolomic changes in WBSCR17 cells	4
Supplementary Figure 4. Metabolomic changes in SLC17A5 cells	6
Supplementary Figure 5. Metabolomic changes in OGDHL-WT cells	7
Supplementary Figure 6. Metabolomic changes in OGDHL-MUT cells	9
Supplementary Figure 7. METIS1 VS METIS2	10
Supplementary Figure 8. Near-significant changes in OGDHL-WT cells	11
Supplementary Table 1. Output from METIS1 search for recurrent mutations	13
Supplementary Table 2. METIS1 mutations in genes associated with metabolic functions	24
Supplementary Table 3. Candidate change-of-metabolic-function mutations from METIS1	30

Supplementary Table 4. Gold standard COMF mutations 31

Supplementary Table 5. Top candidate change of metabolic function mutations from METIS2 33

Supplementary Table 6. Comparison between computational predictors of mutated enzyme function 34

Supplementary Table 7. cDNA sequences for plasmids used in this study 35

Supplementary Notes 43

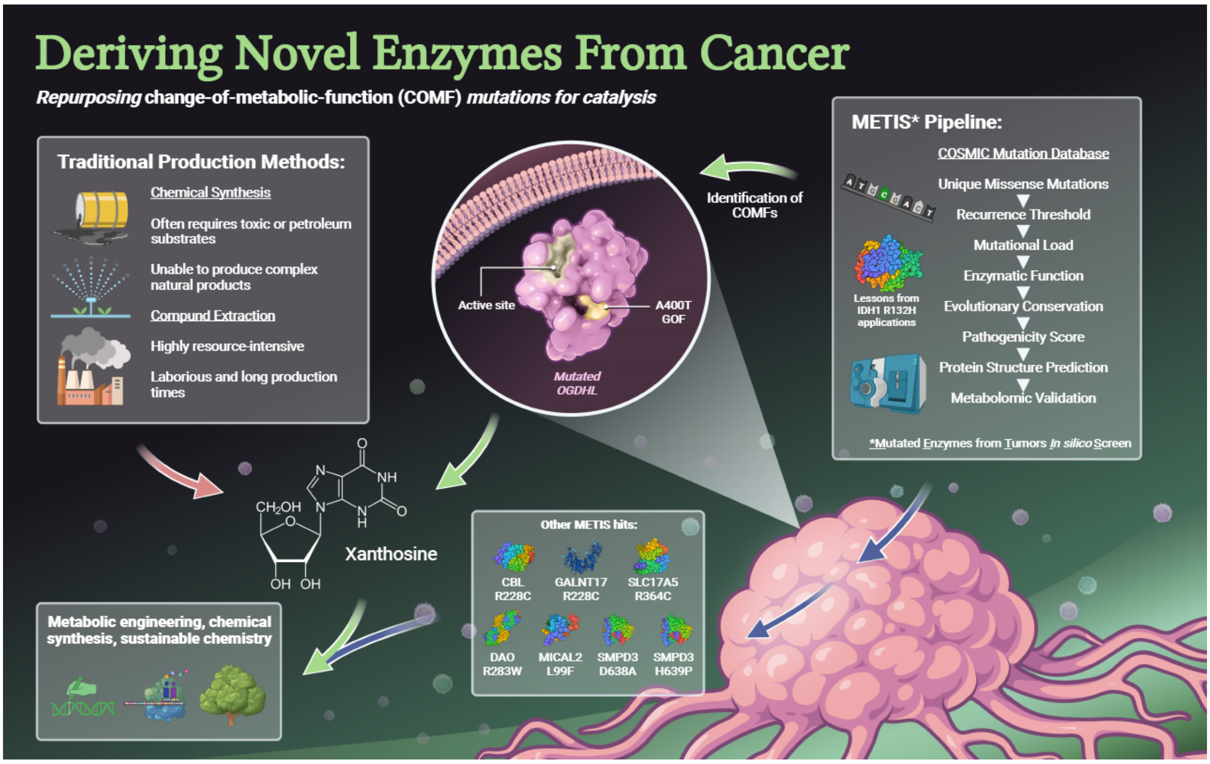

Supplementary Figure 1. Graphical abstract of the METIS pipeline and results.

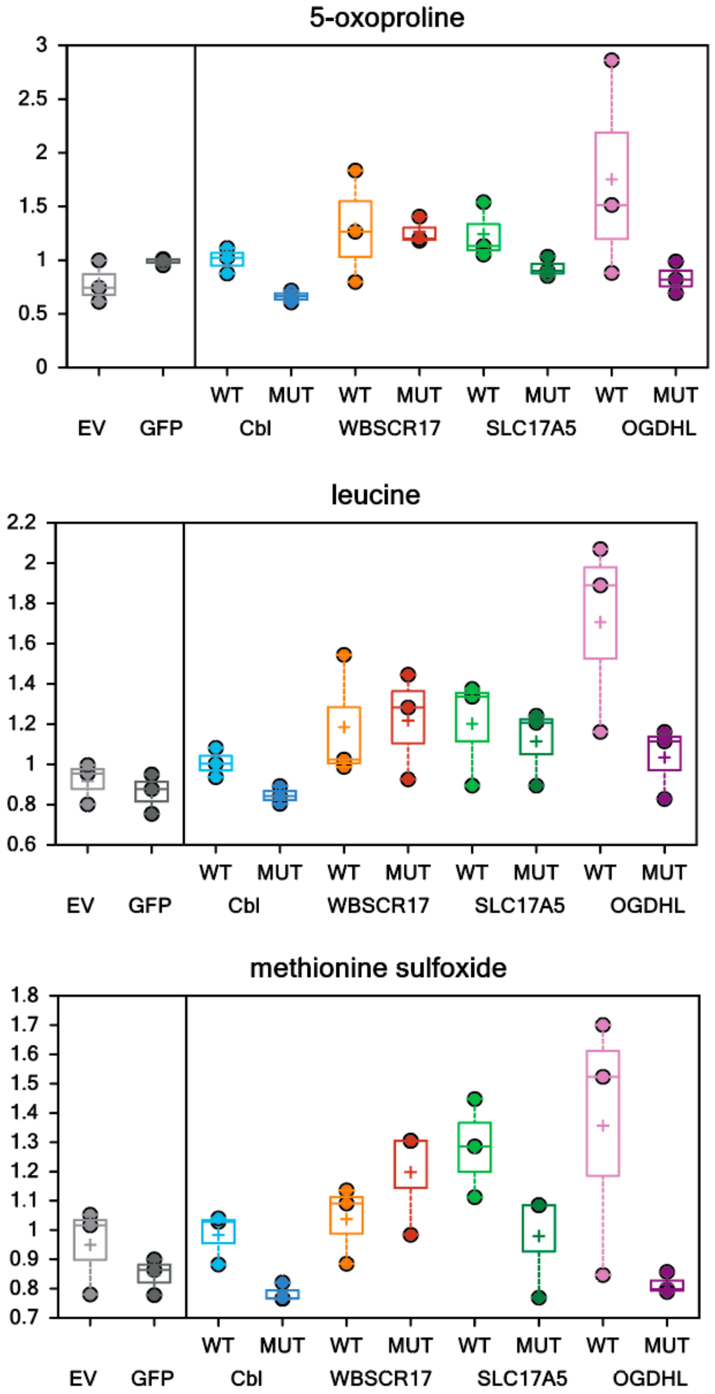

Supplementary Figure 2. Biochemicals reduced in cells expressing CBL mutant vs. CBL WT (P<0.05, Welch's t test).

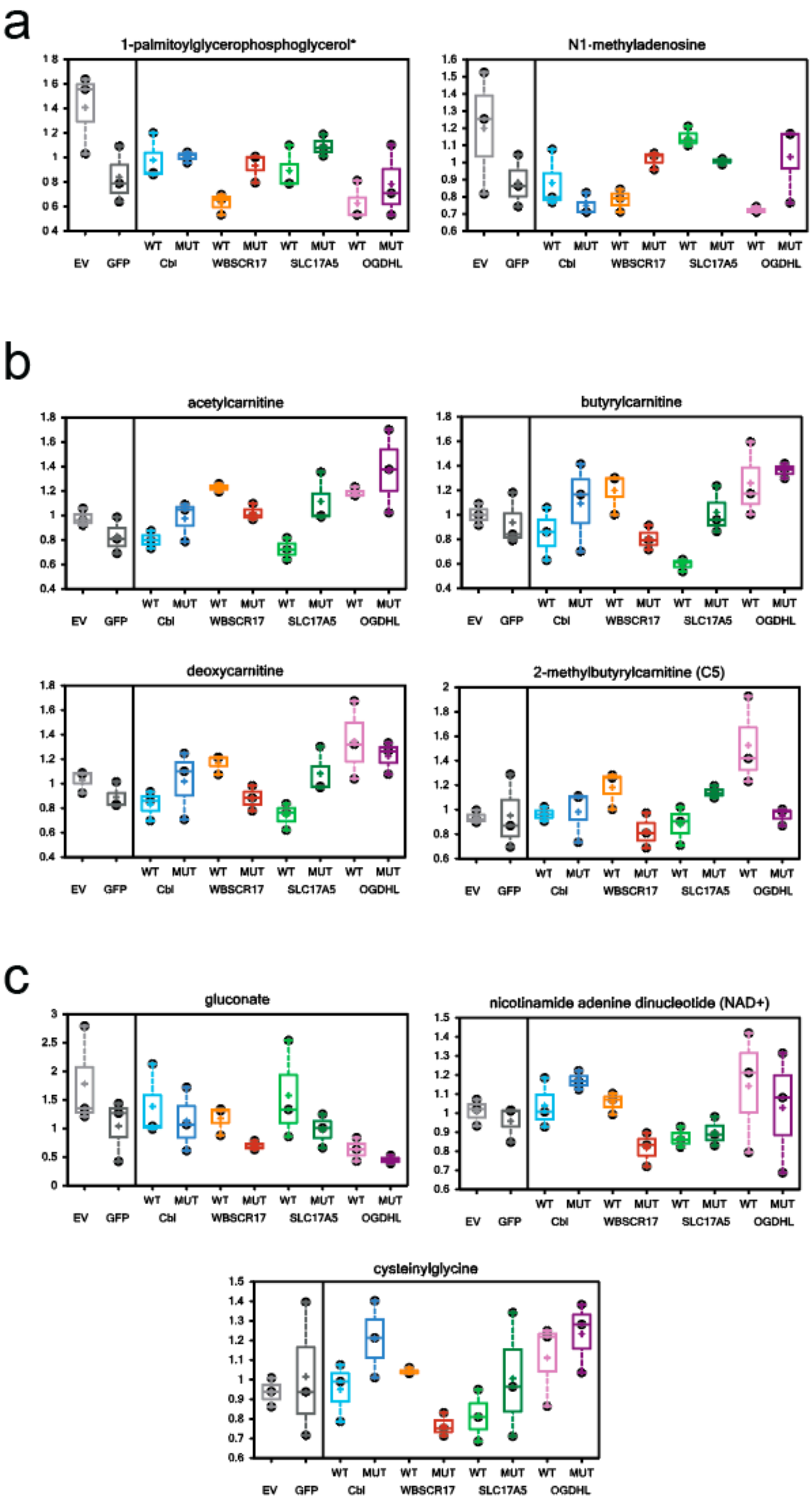
a
1-palmitoylglycerophosphoglycerol*
N1-methyladenosine
b
acetylcarnitine
butyrylcarnitine
deoxycarnitine
2-methylbutyrylcarnitine (C5)
c
gluconate
nicotinamide adenine dinucleotide (NAD+)
cysteinylglycine
WT MUT WT MUT WT MUT WT MUT
EV GFP Cbl WBSCR17 SLC17A5 OGDHL

Supplementary Figure 3. Biochemicals with significant differences in cells expressing WBSCR17 mutant vs. WBSCR17 WT. a. Metabolites increased in mutant vs. WT ($P<0.05$, Welch's t test). **b.** Carnitine metabolites decreased in mutant vs. WT ($P<0.05$, Welch's t test). **c.** Other metabolites decreased in mutant vs. WT ($P<0.05$, Welch's t test)

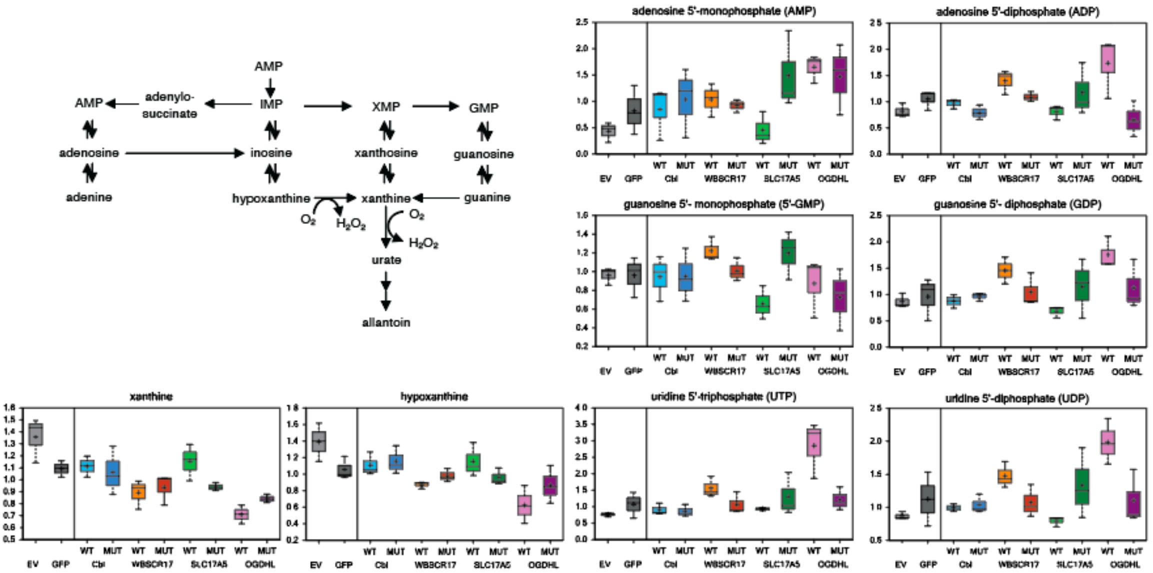

Supplementary Figure 4. Biochemicals altered in cells expressing mutant SLC17A5. SLC17A5 mutants possessed elevated levels of ADP, AMP, GDP, 5'-GMP, UTP, UDP, UMP, CTP, CDP, and CMP compared to wild-type counterparts. These observations may reflect increased availability for DNA and RNA synthesis and potentially result from a decrease in purine and pyrimidine degradation as suggested by lower levels of the catabolic products adenosine, xanthine, hypoxanthine, and uracil.

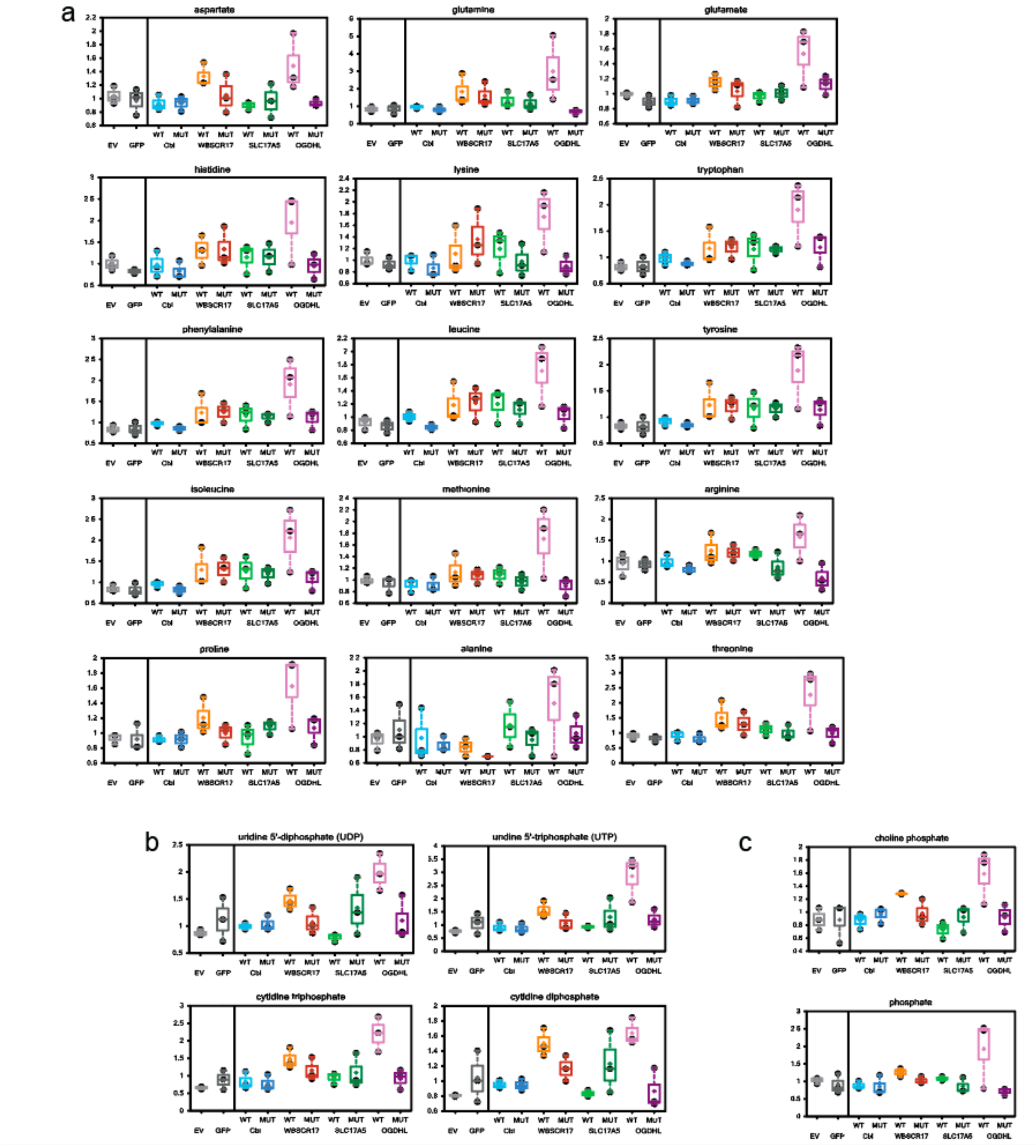

Supplementary Figure 5. Biochemicals increased in cells expressing OGDHL-WT. a. Amino acid metabolites that were significantly different between OGDHL WT and all other samples (Welch's t test with Bonferroni FDR correction q<0.05). **b**. Nucleoside metabolites that were significantly different between OGDHL WT and all other samples (Welch's t test with Bonferroni FDR correction q<0.05). **c**. Other metabolites that were significantly

different between OGDHL WT and all other samples (Welch's t test with Bonferroni FDR correction q<0.05).

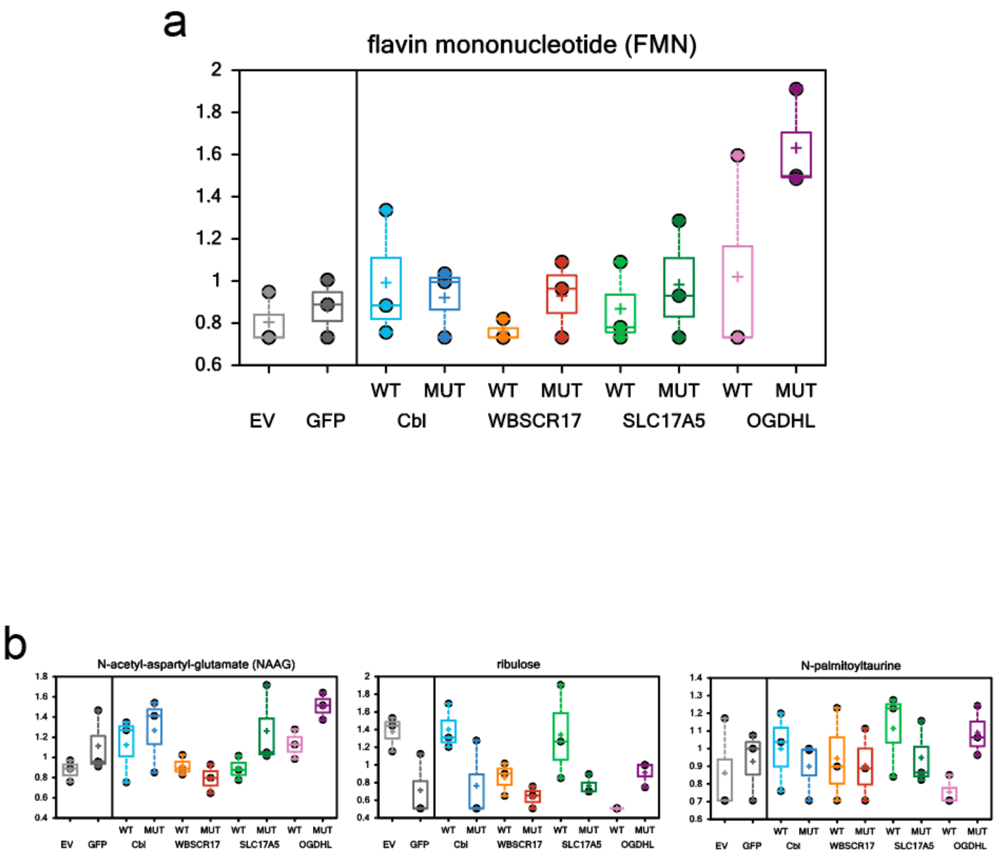

Supplementary Figure 6. Biochemicals increased in cells expressing OGDHL p.A400T. a. Metabolites that were significantly different between the OGDHL mutant and all other groups (Welch's t test with Bonferroni FDR correction q<0.05). **b.** Metabolites that were significantly different between the OGDHL mutant and OGDHL WT (Welch's t test p<0.05).

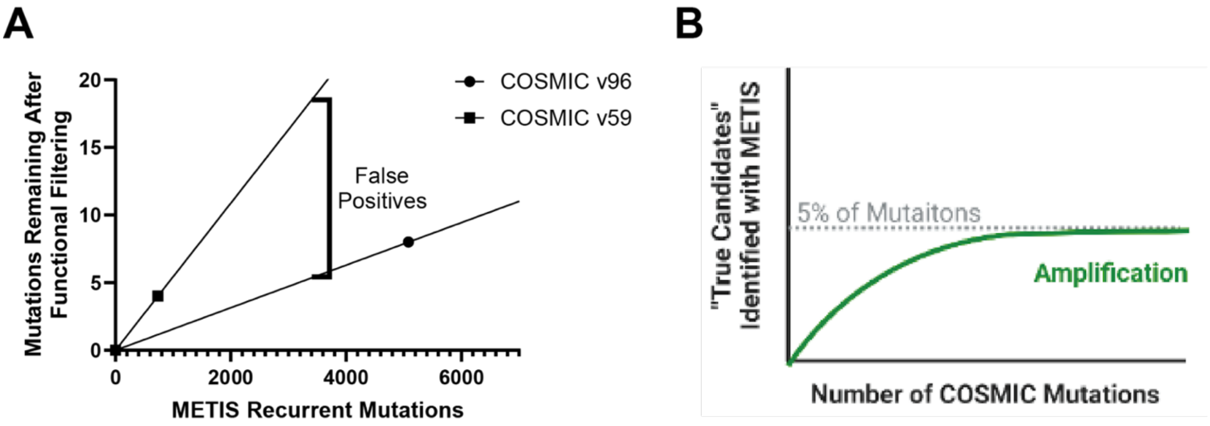

Supplementary Figure 7. Comparison of pipelines to identify COMF mutants from cancer sequencing data. a. Theoretical number of recurrent mutations remaining after conservation and enzyme function filtering between METIS using COSMIC v59 and COSMIC v96 data suggests a lower rate of false positives with more data. **b.** True candidates rise quickly with COSMIC mutations and peak at 5% of switch-of -function mutations.

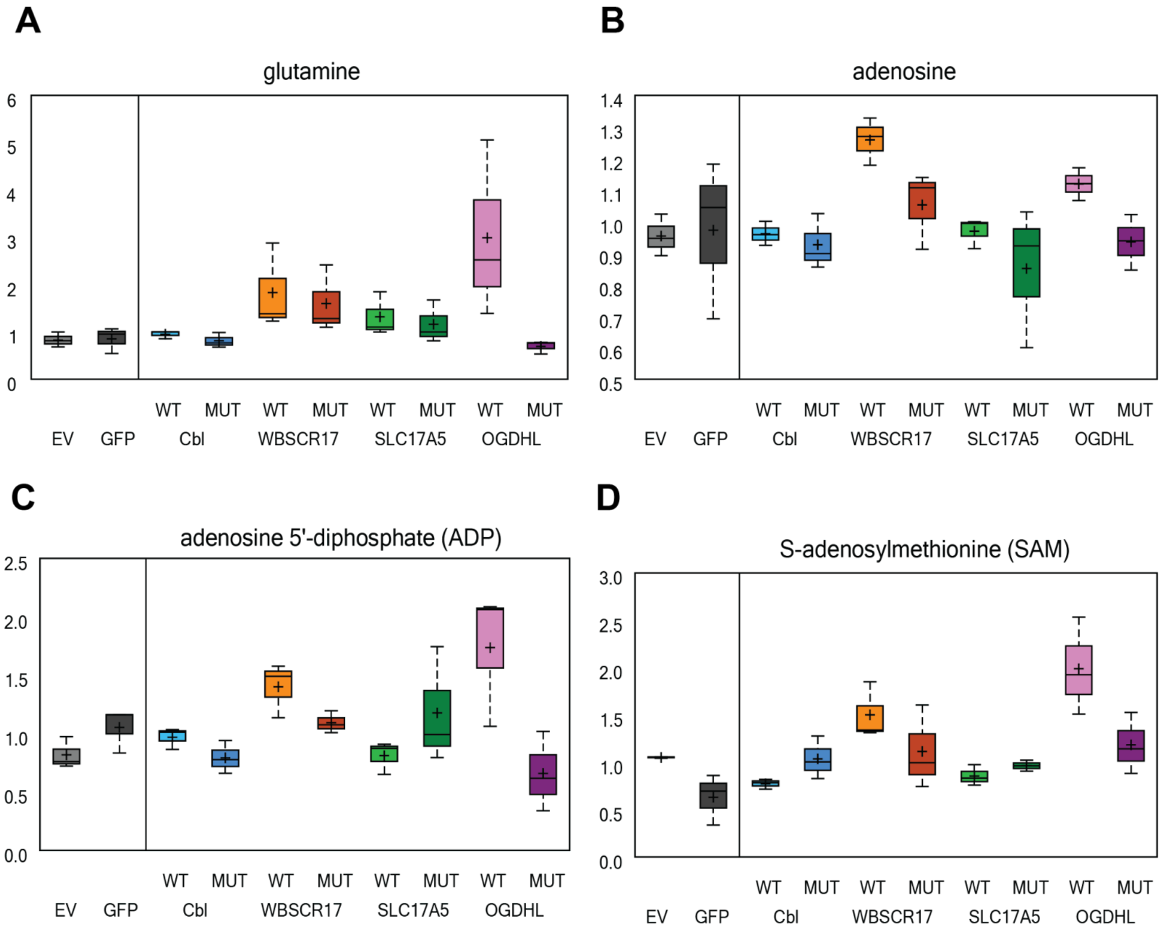

Supplementary Figure 8. Selected biochemicals near-significantly increased in cells expressing OGDHL WT. a. Glutamate, **b.** adenosine, **c.** adenosine diphosphate, **d.** SAM. Metabolites that were near-significantly different between the OGDHL mutant and OGDHL WT (Welch's t test).

Gene	**Gene Name**	**Mut Score**	Total mutations	Most frequent Hotspot	Mutations in most frequent hotspot	Mutations in all hotspots	Number of hotspots	Hotspot mutations
ABL1	c-abl oncogene 1, receptor tyrosine kinase	0.1691259932	881	p.T315I	149	623	19	p.M244V, p.M351T, p.F317L, p.E255K, p.T315I, p.Y253H, p.G250E, p.Y253F, p.E255V, p.F359C, p.F359V, p.F359I, p.Y253C, p.E255L, p.F359Y, p.T315L, p.F317V, p.T315N, p.F359A
ADAMTSL3	ADAMTS-like 3	0.05714285714	35	p.R855C	2	3	2	p.R855C, p.R855H
AKAP12	A kinase (PRKA) anchor protein 12	0.1578947368	19	p.E1282K	3	3	1	p.E1282K
AKT1	v-akt murine thymoma viral oncogene homolog 1	0.9225352113	142	p.E17K	131	131	1	p.E17K
ALK	anaplastic lymphoma receptor tyrosine kinase	0.2317073171	164	p.F1174L	38	105	12	p.F1174L, p.F1174I, p.F1245I, p.F1245L, p.R1275Q, p.F1245V, p.F1245C, p.F1174S, p.R1275?, p.F1174C, p.F1174V, p.R1275L
ANKLE2	ankyrin repeat and LEM domain containing 2	0.4285714286	7	p.P691L	3	3	1	p.P691L
APC	adenomatous polyposis coli	0.08411214953	214	p.S1341R	18	18	1	p.S1341R
ARF4	ADP-ribosylation factor 4	0.5	4	p.R149H	2	3	2	p.R149H, p.R149C
ASXL1	additional sex combs like 1 (Drosophila)	0.2083333333	48	p.E1102D	10	13	2	p.E1102D, p.N986S
ATM	similar to Serine-protein kinase ATM (Ataxia telangiectasia mutated) (A-T, mutated); ataxia telangiectasia mutated	0.05434782609	184	p.D1853N	10	11	2	p.D1853V, p.D1853N
ATP6V1C2	ATPase, H+ transporting, lysosomal 42kDa, V1 subunit C2	0.3333333333	3	p.V118A	1	3	3	p.V118L, p.V118M, p.V118A

AXIN1	axin 1	0.03225806452	124	p.A76T	4	7	2	p.A76V, p.A76T
B2M	beta-2-microglobulin	0.1111111111	18	p.M1K	2	8	5	p.M1K, p.M1R, p.M1T, p.M1V, p.M1L
BRAF	v-raf murine sarcoma viral oncogene homolog B1	0.9511191408	19926	p.V600E	18952	19262	8	p.V600E, p.V600K, p.V600R, p.V600A, p.V600M, p.V600L, p.V600G, p.V600D
C16orf45	chromosome 16 open reading frame 45	1	3	p.T106N	3	3	1	p.T106N
CARD11	caspase recruitment domain family, member 11	0.05555555556	54	p.D223N	3	6	3	p.D223N, p.R416W, p.R416Q
CBL	Cas-Br-M (murine) ecotropic retroviral transforming sequence	0.125	136	p.Y371H	17	72	18	p.C404Y, p.R420Q, p.C384R, p.L380P, p.P417A, p.Y371H, p.R420L, p.P417L, p.C384Y, p.Y371D, p.Y371C, p.C404R, p.P417S, p.R420P, p.C404S, p.R420G, p.P417R, p.Y371S
CCDC41	coiled-coil domain containing 41	0.75	4	p.K208I	3	3	1	p.K208I
CDKN2A	cyclin-dependent kinase inhibitor 2A (melanoma, p16, inhibits CDK4)	0.06054279749	479	p.H83Y	29	93	11	p.H83Y, p.D108Y, p.P114L, p.P114S, p.D108H, p.H83Q, p.H83R, p.H83N, p.D108N, p.P114H, p.H83P
CEBPA	CCAAT/enhancer binding protein (C/EBP), alpha	0.0625	48	p.G340S	3	15	8	p.P39S, p.R300G, p.S21G, p.R300L, p.G340S, p.S21R, p.R297P, p.P39H
CHEK2	protein kinase CHK2-like; CHK2 checkpoint homolog (S. pombe); similar to hCG1983233	0.4444444444	9	p.P536L	4	4	1	p.P536L
CIC	capicua homolog (Drosophila)	0.1818181818	22	p.R215W	4	5	2	p.R215Q, p.R215W
CREBBP	CREB binding protein	0.04255319149	94	p.R1446H	4	9	3	p.R1446H, p.R1446L, p.R1446C
CRLF2	cytokine receptor-like factor 2	1	10	p.F232C	10	10	1	p.F232C

CSF1R	colony stimulating factor 1 receptor	0.2954545455	44	p.Y969C	13	32	9	p.G413S, p.Y969C, p.Y969F, p.L301V, p.L301F, p.L301S, p.Y969N, p.Y969D, p.Y969H
CSMD1	CUB and Sushi multiple domains 1	0.2222222222	18	p.E616K	4	12	3	p.T1464R, p.E616K, p.Q2270R
CTNNB1	catenin (cadherin-associated protein), beta 1, 88kDa	0.1831701124	2757	p.T41A	505	2469	36	p.T41I, p.D32Y, p.G34E, p.S33C, p.T41A, p.S45F, p.S37F, p.G34V, p.D32N, p.D32V, p.S33Y, p.S37Y, p.S37C, p.S33F, p.D32A, p.S45A, p.D32H, p.S33A, p.S33P, p.S45P, p.D32G, p.G34R, p.S37P, p.D32E, p.S37A, p.S33L, p.S45Y, p.S33N, p.S33T, p.S45C, p.T41P, p.S45E, p.T41S, p.T41N, p.S37T, p.S45T
CYP19A1	cytochrome P450, family 19, subfamily A, polypeptide 1	0.2	10	p.P8L	2	3	2	p.P8L, p.P8S
CYP4A11	cytochrome P450, family 4, subfamily A, polypeptide 11	0.3	10	p.V185F	3	3	1	p.V185F
DCC	deleted in colorectal carcinoma	0.1153846154	26	p.G55E	3	3	1	p.G55E
DNMT3A	DNA (cytosine-5-)-methyltransferase 3 alpha	0.4631578947	475	p.R882H	220	383	7	p.R882H, p.R882C, p.R882P, p.R882S, p.R882L, p.?, p.R882G
EGFR	epidermal growth factor receptor (erythroblastic leukemia viral (v-erb-b) oncogene homolog, avian)	0.7251563586	5756	p.L858R	4174	4645	9	p.L858R, p.T790M, p.L858A, p.L858W, p.L858K, p.L858Q, p.L858G, p.L858P, p.L858M
ERBB2	v-erb-b2 erythroblastic leukemia viral oncogene homolog 2, neuro/glioblastoma derived oncogene homolog (avian)	0.08219178082	73	p.L755S	6	24	10	p.V777L, p.L755P, p.S310Y, p.G776L, p.S310F, p.L755S, p.V777M, p.G776V, p.G776S, p.V777A
ERBB3	v-erb-b2 erythroblastic leukemia viral oncogene homolog 3, neuro/glioblastoma derived oncogene homolog (avian)	0.15	20	p.V104M	3	5	2	p.V104L, p.V104M

ERCC6	excision repair cross-complementing rodent repair deficiency, complementation group 6	0.04545454545	22	p.E1119L	1	3	3	p.E1119Q, p.E1119V, p.E1119L
EZH2	enhancer of zeste homolog 2 (Drosophila)	0.2487309645	197	p.Y646F	49	126	6	p.Y646F, p.Y646N, p.Y646S, p.Y646C, p.Y646H, p.Y646?
FBXW7	F-box and WD repeat domain containing 7	0.2068965517	261	p.R465C	54	179	13	p.R465C, p.R465H, p.R479G, p.R479Q, p.R505C, p.R479L, p.R505L, p.R505H, p.R465L, p.R505S, p.R465Y, p.R505G, p.R505P
FGFR2	fibroblast growth factor receptor 2	0.2043010753	93	p.S252W	19	26	2	p.S252W, p.N549K
FGFR3	fibroblast growth factor receptor 3	0.5436193223	2774	p.S249C	1508	2446	9	p.S249C, p.Y373C, p.R248C, p.K650Q, p.K650E, p.K650M, p.K650T, p.S249T, p.K650N
FKBP9	FK506 binding protein 9, 63 kDa	0.6	10	p.R107H	6	6	1	p.R107H
FLT3	fms-related tyrosine kinase 3	0.5571847507	1023	p.D835?	570	927	9	p.D835?, p.D835V, p.D835Y, p.D835H, p.D835E, p.D835N, p.D835A, p.D835I, p.D835F
FOXL2	forkhead box L2	0.996978852	331	p.C134W	330	330	1	p.C134W
GATA1	GATA binding protein 1 (globin transcription factor 1)	0.1875	32	p.M1I	6	23	9	p.V74I, p.M1N, p.M1V, p.M1I, p.V74D, p.M1R, p.M1T, p.M1L, p.V74F
GATA2	GATA binding protein 2	0.5714285714	14	p.L359V	8	8	1	p.L359V
GNA11	guanine nucleotide binding protein (G protein), alpha 11 (Gq class)	0.8725490196	102	p.Q209L	89	97	4	p.Q209L, p.R183H, p.Q209P, p.R183C
GNAQ	guanine nucleotide binding protein (G protein), q polypeptide	0.4954545455	220	p.Q209P	109	206	6	p.Q209L, p.Q209P, p.Q209R, p.Q209Y, p.Q209H, p.Q209K
GNAS	GNAS complex locus	0.6339522546	377	p.R201C	239	370	9	p.Q227H, p.R201H, p.R201C, p.Q227R, p.Q227L, p.R201S, p.Q227E, p.Q227K, p.R201L

GRIN2A	glutamate receptor, ionotropic, N-methyl D-aspartate 2A	0.05769230769	52	p.S278F	3	3	1	p.S278F
GSTM5	glutathione S-transferase mu 5	1	4	p.L179P	4	4	1	p.L179P
HLA-E	major histocompatibility complex, class I, E	0.5	6	p.W346S	3	3	1	p.W346S
HLCS	holocarboxylase synthetase (biotin-(proprionyl-Coenzyme A-carboxylase (ATP-hydrolysing)) ligase)	0.3333333333	9	p.E362K	3	3	1	p.E362K
HNF1A	HNF1 homeobox A	0.12	75	p.W206C	9	31	9	p.W206L, p.N237S, p.W206C, p.R263H, p.R272H, p.R263C, p.R272S, p.R263L, p.N237K
HRAS	v-Ha-ras Harvey rat sarcoma viral oncogene homolog	0.3276089828	757	p.G12V	248	739	17	p.G12V, p.G13R, p.G12S, p.G12C, p.G13V, p.Q61R, p.Q61L, p.Q61K, p.G12R, p.G12D, p.G13C, p.G13D, p.G13S, p.Q61H, p.G12A, p.Q61P, p.Q61E
IDH1	isocitrate dehydrogenase 1 (NADP+), soluble	0.7140943194	3732	p.R132H	2665	3696	7	p.R132C, p.R132H, p.R132S, p.R132G, p.R132L, p.R132V, p.R132?
IDH2	isocitrate dehydrogenase 2 (NADP+), mitochondrial	0.6670317634	913	p.R140Q	609	894	9	p.R172G, p.R172M, p.R172K, p.R172W, p.R172S, p.R140Q, p.R140L, p.R140W, p.R140G
IL7R	interleukin 7 receptor	0.2105263158	19	p.S185C	4	4	1	p.S185C
JAK1	Janus kinase 1	0.1509433962	53	p.R724H	8	20	7	p.V658F, p.R724H, p.R879S, p.R879C, p.R879H, p.Y652H, p.R724Q
JAK2	Janus kinase 2	0.9942608811	30144	p.V617F	29971	29972	2	p.V617F, p.V617I
JAK3	Janus kinase 3	0.1774193548	62	p.R657Q	11	22	3	p.Q501H, p.A573V, p.R657Q
KDR	kinase insert domain receptor (a type III receptor tyrosine kinase)	0.05555555556	36	p.T771R	2	3	2	p.T771R, p.T771M

KIT	similar to Mast/stem cell growth factor receptor precursor (SCFR) (Proto-oncogene tyrosine-protein kinase Kit) (c-kit) (CD117 antigen); v-kit Hardy-Zuckerman 4 feline sarcoma viral oncogene homolog	0.3663031215	2659	p.D816V	974	1717	20	p.V560D, p.D816V, p.V559D, p.V559G, p.V560G, p.D816I, p.D816H, p.D816G, p.D816F, p.D816Y, p.D816?, p.D816E, p.V560E, p.V559A, p.V559I, p.D816A, p.V560A, p.V559K, p.D816N, p.V559F
KLHL4	kelch-like 4 (Drosophila)	0.1111111111	18	p.R57W	2	3	2	p.R57W, p.R57Q
KRAS	v-Ki-ras2 Kirsten rat sarcoma viral oncogene homolog	0.341255625	22889	p.G12D	7811	22347	22	p.G12V, p.G12D, p.G12A, p.G12S, p.G12R, p.G12C, p.G13D, p.G13C, p.G13A, p.G13S, p.G12F, p.G13V, p.G12Y, p.G13R, p.G13E, p.G12E, p.G12I, p.G12L, p.G12W, p.G13N, p.G12N, p.G13I
LOC652737		0.07692307692	13	p.T151L	1	3	3	p.T151M, p.T151P, p.T151L
LPAR4	lysophosphatidic acid receptor 4	0.1	10	p.D307H	1	3	3	p.D307N, p.D307H, p.D307Y
MAG	myelin associated glycoprotein	0.2307692308	13	p.L202M	3	3	1	p.L202M
MAP2K4	mitogen-activated protein kinase kinase 4	0.1111111111	27	p.S184L	3	6	3	p.R134Q, p.S184L, p.R134W
MED12	mediator complex subunit 12	0.3754512635	277	p.G44D	104	255	7	p.G44A, p.G44S, p.G44D, p.G44R, p.G44V, p.G44C, p.L36R
MEN1	multiple endocrine neoplasia I	0.03389830508	59	p.W183R	2	3	2	p.W183R, p.W183G
MET	met proto-oncogene (hepatocyte growth factor receptor)	0.3381294964	139	p.Y1253D	47	85	5	p.T1010I, p.Y1253D, p.N375S, p.Y1248H, p.Y1248C
MGA	MAX gene associated	0.1578947368	19	p.R2484Q	3	4	2	p.R2484Q, p.R2484W

MLH1	mutL homolog 1, colon cancer, nonpolyposis type 2 (E. coli)	0.09090909091	33	p.V384D	3	3	1	p.V384D
MPL	myeloproliferative leukemia virus oncogene	0.6060606061	462	p.W515L	280	428	6	p.W515L, p.W515K, p.W515A, p.W515R, p.W515?, p.W515S
MSH6	mutS homolog 6 (E. coli)	0.08	50	p.T1219I	4	4	1	p.T1219I
MYD88	myeloid differentiation primary response gene (88)	0.7027027027	111	p.L265P	78	89	2	p.S219C, p.L265P
MYO3A	myosin IIIA	0.04545454545	44	p.N525S	2	4	3	p.N525K, p.N525H, p.N525S
NF1	neurofibromin 1	0.03896103896	77	p.K1444E	3	5	3	p.K1444E, p.K1444Q, p.K1444N
NF2	neurofibromin 2 (merlin)	0.1	40	p.V219M	4	4	1	p.V219M
NFE2L2	nuclear factor (erythroid-derived 2)-like 2	0.1011235955	89	p.E79K	9	75	28	p.R34Q, p.D77G, p.E79Q, p.D29Y, p.E79K, p.D29H, p.E82Q, p.D77A, p.G81D, p.D77N, p.E79G, p.G81V, p.E82V, p.T80K, p.W24R, p.W24C, p.D29G, p.L30F, p.D77V, p.T80P, p.L30R, p.R34G, p.R34P, p.T80I, p.D29N, p.E82D, p.T80R, p.E82G
NOS1	nitric oxide synthase 1 (neuronal)	0.09523809524	21	p.S771L	2	0	0	p.S771L
NOTCH1	Notch homolog 1, translocation-associated (Drosophila)	0.09905660377	424	p.L1601P	42	213	11	p.L1575P, p.L1586P, p.L1594P, p.R1599P, p.L1601P, p.L1679P, p.L1679Q, p.L1586Q, p.L1575Q, p.L1601Q, p.L1586R
NOTCH4	Notch homolog 4 (Drosophila)	0.08333333333	24	p.E1977Q	2	3	2	p.E1977Q, p.E1977K
NRAS	neuroblastoma RAS viral (v-ras) oncogene homolog	0.2936235204	2619	p.Q61R	769	2567	25	p.G12A, p.G12V, p.Q61R, p.Q61H, p.G13D, p.G12D, p.Q61K, p.G13R, p.Q61L, p.G12S, p.G12R, p.G12C, p.G13N, p.G13C, p.G13V, p.G13A,

								p.Q61E, p.G13S, p.G12T, p.Q61P, p.G12E, p.G12Y, p.G12P, p.G13Y, p.G12N
NTRK3	neurotrophic tyrosine kinase, receptor, type 3	0.04545454545	44	p.R731Q	2	4	3	p.R731P, p.R731Q, p.R731W
NUP93	nucleoporin 93kDa	0.3333333333	9	p.E14K	3	3	1	p.E14K
ODZ1	odz, odd Oz/ten-m homolog 1(Drosophila)	0.03703703704	54	p.A2260T	2	6	4	p.A2260T, p.R2551G, p.A2260V, p.R2551W
OGDHL	oxoglutarate dehydrogenase like	0.02542372881	118	p.A400T	3	3	1	p.A400T
PAX5	paired box 5	0.5	38	p.P80R	19	22	2	p.V26G, p.P80R
PCDH18	protocadherin 18	0.09523809524	21	p.R582H	2	3	2	p.R582C, p.R582H
PCDHB12	protocadherin beta 12	0.2307692308	13	p.V392M	3	3	1	p.V392M
PDGFRA	platelet-derived growth factor receptor, alpha polypeptide	0.6810699588	486	p.D842V	331	383	8	p.D842V, p.V561D, p.D842F, p.D842I, p.D842Y, p.V561A, p.D842H, p.D842A
PIK3CA	phosphoinositide-3-kinase, catalytic, alpha polypeptide	0.3650749584	3602	p.H1047R	1315	2927	16	p.E545K, p.E542K, p.H1047L, p.H1047R, p.E545G, p.E545A, p.E545D, p.E542V, p.E545Q, p.H1047Q, p.H1047Y, p.E542Q, p.(542_545)E>K, p.E545V, p.H1047T, p.E542G
PIK3R1	phosphoinositide-3-kinase, regulatory subunit 1 (alpha)	0.1176470588	34	p.G376R	4	12	5	p.R574T, p.N564D, p.G376R, p.R574I, p.N564K
POSTN	periostin, osteoblast specific factor	0.3333333333	9	p.R508C	3	3	1	p.R508C
PPP2R1A	protein phosphatase 2 (formerly 2A), regulatory subunit A, alpha isoform	0.2714285714	70	p.R183W	19	60	10	p.R183W, p.S256F, p.R183Q, p.S256Y, p.R182W, p.P179R,

								p.W257G, p.R183G, p.W257C, p.P179L
PRKCI	protein kinase C, iota	0.2727272727	11	p.R471C	3	3	1	p.R471C
PTEN	phosphatase and tensin homolog; phosphatase and tensin homolog pseudogene 1	0.0875331565	754	p.R130G	66	183	7	p.R130G, p.R173H, p.R173C, p.R130Q, p.R130L, p.R130P, p.R173P
PTPN11	protein tyrosine phosphatase, non-receptor type 11; similar to protein tyrosine phosphatase, non-receptor type 11	0.1811414392	403	p.E76K	73	323	22	p.D61Y, p.G60V, p.D61N, p.D61H, p.E76K, p.A72T, p.D61V, p.A72D, p.E76G, p.E76Q, p.E76V, p.A72V, p.E76A, p.D61G, p.G503A, p.G503V, p.G60R, p.G503E, p.G60A, p.G503R, p.G503L, p.E76M
RB1	retinoblastoma 1	0.075	40	p.R661W	3	3	1	p.R661W
RET	ret proto-oncogene	0.7398190045	442	p.M918T	327	364	6	p.M918T, p.C634R, p.C634W, p.C634Y, p.C634A, p.C634T
RSPO2	R-spondin 2 homolog (Xenopus laevis)	0.3333333333	9	p.R28C	3	3	1	p.R28C
RUNX1	runt-related transcription factor 1	0.09356725146	171	p.R201Q	16	77	11	p.R107C, p.D198N, p.R166G, p.L56S, p.R107H, p.D198G, p.D198V, p.R201Q, p.R166P, p.R166Q, p.R201G
SF3B1	splicing factor 3b, subunit 1, 155kDa	0.5726681128	461	p.K700E	264	387	15	p.K700E, p.K666E, p.H662D, p.H662Q, p.K666M, p.R625L, p.R625C, p.K666N, p.H662Y, p.K666Q, p.R625H, p.R625G, p.K666R, p.K666T, p.R625P
SLC17A5	solute carrier family 17 (anion/sugar transporter), member 5	0.375	8	p.R364C	3	3	1	p.R364C
SLC24A4	solute carrier family 24 (sodium/potassium/calcium exchanger), member 4	0.5	4	p.V422I	2	3	2	p.V422I, p.V422D
SMAD4	SMAD family member 4	0.05147058824	136	p.D351H	7	22	5	p.R361C, p.D351H, p.R361H, p.R361S, p.D351N

SMARCB1	SWI/SNF related, matrix associated, actin dependent regulator of chromatin, subfamily b, member 1	0.1666666667	24	p.R377H	4	4	1	p.R377H
SMO	smoothened homolog (Drosophila)	0.3225806452	31	p.W535L	10	10	1	p.W535L
SRSF2		0.4935064935	77	p.P95H	38	74	4	p.P95H, p.P95L, p.P95R, p.P95?
STK11	serine/threonine kinase 11	0.2626262626	99	p.F354L	26	42	6	p.D194Y, p.D194V, p.F354L, p.P281L, p.D194H, p.D194N
TAF4	TAF4 RNA polymerase II, TATA box binding protein (TBP)-associated factor, 135kDa	0.2857142857	7	p.R459Q	2	3	2	p.R459G, p.R459Q
TCF7L2	transcription factor 7-like 2 (T-cell specific, HMG-box)	0.3	10	p.R465C	3	3	1	p.R465C
TET2	tet oncogene family member 2	0.05762711864	295	p.I1873T	17	18	2	p.I1873T, p.I1873N
TGFBR2	transforming growth factor, beta receptor II (70/80kDa)	0.08333333333	24	p.D446N	2	3	2	p.D446N, p.D446V
TP53	tumor protein p53	0.06234351527	11982	p.R175H	747	2914	19	p.R248Q, p.R248W, p.R175H, p.R273C, p.R248P, p.R273H, p.R273L, p.R273P, p.R175L, p.R175C, p.R273S, p.R248L, p.R175S, p.R248G, p.R175G, p.R273G, p.R248C, p.R175P, p.R248Y
TRIM23	tripartite motif-containing 23	0.3333333333	9	p.R289Q	3	3	1	p.R289Q
TRRAP	transformation/transcription domain-associated protein	0.1111111111	45	p.S722F	5	5	1	p.S722F
TSHR	thyroid stimulating hormone receptor	0.146179402	301	p.T632I	44	186	15	p.D619G, p.A623S, p.A623V, p.T632I, p.D633Y, p.D633H, p.F631L, p.D633E, p.M453T, p.F631I, p.T632A, p.F631C, p.F631V, p.A623I, p.A623F

U2AF1	U2 small nuclear RNA auxiliary factor 1	0.4736842105	76	p.S34F	36	71	4	p.S34F, p.S34Y, p.Q157P, p.Q157R
WBSCR17	Williams-Beuren syndrome chromosome region 17	0.1764705882	17	p.R228C	3	3	1	p.R228C
WNK1	WNK lysine deficient protein kinase 1; hypothetical LOC100132369	0.12	25	p.I1172M	3	3	1	p.I1172M
WT1	Wilms tumor 1	0.1126760563	71	p.D396N	8	28	7	p.R394W, p.R394G, p.R394P, p.D396H, p.R394Q, p.D396N, p.D396G
XPO1	exportin 1 (CRM1 homolog, yeast)	0.1818181818	11	p.E571K	2	3	2	p.E571K, p.E571V
ZNF708	zinc finger protein 708	0.3	10	p.N39D	3	3	1	p.N39D
ZNF831	zinc finger protein 831	0.1666666667	18	p.S1474F	3	3	1	p.S1474F

Supplementary Table 1. Output from METIS1 search for recurrent mutations

Gene	**Gene Name**	Most frequent Hotspot	**EC Number**	**Previously unknown GOF?**	**Available structure ?**	**Enzyme Domain ?**	**Enzyme Active Site?**	**Conserved Residue?**	**>1000 residues ?**	**Truncating mutational distribution ?**	**Pseudogene interference ?**	**Inactivating mutation spectrum?**
GSTM5	glutathione S-transferase mu 5	p.L179P	2.5.1.18,								X	
JAK2	Janus kinase 2	p.V617F	2.7.10.2,	X								
BRAF	v-raf murine sarcoma viral oncogene homolog B1	p.V600E	2.7.11.1,	X								
AKT1	v-akt murine thymoma viral oncogene homolog 1	p.E17K	2.7.11.1,	X			X					
RET	ret proto-oncogene	p.M918T	2.7.10.1,	X								
EGFR	epidermal growth factor receptor (erythroblastic leukemia viral (v-erb-b) oncogene homolog, avian)	p.L858R	2.7.10.1,	X								
IDH1	isocitrate dehydrogenase 1 (NADP+), soluble	p.R132H	1.1.1.42,	X								
PDGFRA	platelet-derived growth factor receptor, alpha polypeptide	p.D842V	2.7.10.1,	X								

IDH2	isocitrate dehydrogenase 2 (NADP+), mitochondrial	p.R140Q	1.1.1.42,	X								
FKBP9	FK506 binding protein 9, 63 kDa	p.R107H	5.2.1.8,								X	
FLT3	fms-related tyrosine kinase 3	p.D835?	2.7.10.1,	X								
FGFR3	fibroblast growth factor receptor 3	p.S249C	2.7.10.1,	X								
DNMT3A	DNA (cytosine-5-)-methyltransferase 3 alpha	p.R882H	2.1.1.37,				X					
CHEK2	protein kinase CHK2-like; CHK2 checkpoint homolog (S. pombe); similar to hCG1983233	p.P536L	2.7.11.1,	X								
SLC17A5	solute carrier family 17 (anion/sugar transporter), member 5	p.R364C	Solute carrier*									
KIT	similar to Mast/stem cell growth factor receptor precursor (SCFR) (Proto-oncogene tyrosine-protein kinase Kit) (c-kit) (CD117 antigen); v-kit Hardy-Zuckerman 4	p.D816V	2.7.10.1,	X								

	feline sarcoma viral oncogene homolog											
PIK3CA	phosphoinositide-3-kinase, catalytic, alpha polypeptide	p.H1047 R	2.7.1.153,	X								
MET	met proto-oncogene (hepatocyte growth factor receptor)	p.Y1253 D	2.7.10.1,	X								
HLCS	holocarboxylase synthetase (biotin-(proprionyl-Coenzyme A-carboxylase (ATP-hydrolysing)) ligase)	p.E362K	6.3.4.9		X							
CYP4A11	cytochrome P450, family 4, subfamily A, polypeptide 11	p.V185F	1.14.15.3,				X	X				
PRKCI	protein kinase C, iota	p.R471C	2.7.11.13,	X								
PPP2R1A	protein phosphatase 2 (formerly 2A), regulatory subunit A, alpha isoform	p.R183W	3.1.3.16,	X								
STK11	serine/threonine kinase 11	p.F354L	2.7.11.1,	X								

EZH2	enhancer of zeste homolog 2 (Drosophila)	p.Y646F	2.1.1.43,	X								
ALK	anaplastic lymphoma receptor tyrosine kinase	p.F1174 L	2.7.10.1,	X								
FGFR2	fibroblast growth factor receptor 2	p.S252W	2.7.10.1,	X								
CYP19A1	cytochrome P450, family 19, subfamily A, polypeptide 1	p.P8L	1.14.14.1,									X
PTPN11	protein tyrosine phosphatase, non-receptor type 11; similar to protein tyrosine phosphatase, non-receptor type 11	p.E76K	3.1.3.48,	X								
JAK3	Janus kinase 3	p.R657Q	2.7.10.2,	X								
WBSCR1 7	Williams-Beuren syndrome chromosome region 17	p.R228C	2.4.1.41,									
ABL1	c-abl oncogene 1, receptor tyrosine kinase	p.T315I	2.7.10.2,	X								
JAK1	Janus kinase 1	p.R724H	2.7.10.2,	X								
ERBB3		p.V104M	2.7.10.1,	X								

CBL	Cas-Br-M (murine) ecotropic retroviral transforming sequence	p.Y371H	6.3.2.-,									
WNK1	WNK lysine deficient protein kinase 1; hypothetical LOC100132369	p.I1172 M	2.7.11.1,									
MAP2K4	mitogen-activated protein kinase kinase 4	p.S184L	2.7.12.2,	X								
NOS1	nitric oxide synthase 1 (neuronal)	p.S771L	1.14.13.39						X			Maybe
PTEN	phosphatase and tensin homolog; phosphatase and tensin homolog pseudogene 1	p.R130G	3.1.3.16, 3.1.3.48, 3.1.3. 67,	X						X		
TGFBR2	transforming growth factor, beta receptor II (70/80kDa)	p.D446N	2.7.11.30,									
ERBB2	v-erb-b2 erythroblastic leukemia viral oncogene homolog 2, neuro/glioblasto ma derived oncogene homolog (avian)	p.L755S	2.7.10.1,	X								
TET2	tet oncogene family member 2	p.I1873T	1.14.11.n2,	X								X

KDR	kinase insert domain receptor (a type III receptor tyrosine kinase)	p.T771R	2.7.10.1,	X								
ATM	similar to Serine-protein kinase ATM (Ataxia telangiectasia mutated) (A-T, mutated); ataxia telangiectasia mutated	p.D1853N	2.7.11.1,	X								
NTRK3	neurotrophic tyrosine kinase, receptor, type 3	p.R731Q	2.7.10.1,	X								
MYO3A	myosin IIIA	p.N525S	2.7.11.1,				X					
ERCC6	excision repair cross-complementing rodent repair deficiency, complementation group 6	p.E1119L	3.6.1.-,	X					X			
CREBBP	CREB binding protein	p.R1446H	2.3.1.48,	X								X
OGDHL	Oxoglutarate dehydrogenase like	p.A400T	1.2.4.2									

Supplementary Table 2. METIS1 mutations in genes associated with metabolic functions

**Gene**	**Gene Name**	**Enzyme Activity**	**Most frequent Hotspot**	cDNA change	COSMIC Mutation ID	Genomic coordinates (GRCh37)
CBL	Cas-Br-M (murine) ecotropic retroviral transforming sequence	E3 Ubiquitin Ligase	p.Y371H	c.1111T>C	COSM34052	chr11:119148891
WBSCR17	Williams-Beuren syndrome chromosome region 17	Protein-UDP acetylgalactosaminyltransferase.	p.R228C	c.682C>T	COSM106581	chr7:70880967
SLC17A5	solute carrier family 17 (anion/sugar transporter), member 5	Sialic acid/proton solute transporter	p.R364C	c.1090C>T	COSM106992	chr6: 6:74325059
OGHDL	oxoglutarate dehydrogenase like	a-ketoglutarate dehydrogenase	p.A400T	c.1198G>A	COSM174929	chr10:50954894

Supplementary Table 3. Candidate change-of-metabolic-function mutations from METIS1

Gene	Pt Mutation	Citation
JAK2	p.V617F	Kralovics, 2005
BRAF	p.V600E	Cantwell-Dorris, 2011
AKT1	p.E17K	Bleeker, 2008
RET	p.M918T	Santoro, 2004
EGFR	p.L858R	Lynch, 2004
IDH1	p.R132H	Reitman, 2014
PDGFRA	p.D842V	Hirota, 2003
IDH2	p.R140Q	Reitman, 2010
FLT3	p.D835?	Yamamoto, 2001
FGFR3	p.S249C	Tomlinson, 2007
KIT	p.D816V	Longley, 1999
PIK3CA	p.H1047R	Samuels, 2004
MET	p.Y1253D	Ma, 2008
PRKCI	p.R471C	Linch, 2013
PPP2R1A	p.R183W	Shih, 2011
STK11	p.F354L	Launonen, 2005
EZH2	p.Y646F	Souroullas, 2016
ALK	p.F1174L	George, 2008
FGFR2	p.S252W	Dutt, 2008
PTPN11	p.E76K	Liu, 2016
JAK3	p.R657Q	Sato, 2008
ABL1	p.T315I	Azam, 2008
JAK1	p.R724H	Flex, 2008
ERBB3	p.V104M	Jaiswal, 2013
MAP2K4	p.S184L	Ellis, 2012
ERBB2	p.L755S	Xu, 2017
KDR	p.T771R	Antonescu, 2009

ATM	p.D1853N	Navrkalova. 2013
NTRK3	p.R731Q	Jin, 2020
CREBBP	p.R1446H	Mullighan, 2011
DNMT3A	p.R882H	Russler-Germain, 2014
CYP19A1	p.P8L	Fukami, 2011
CBL	p.Y371H	Fernandes 2010
WNK1	p.I1172M	Sie, 2020
ABL1	E255K	Quintás-Cardama 2009
AKT2	E17K	Hussain, 2011
CDK4	R24C	Rane 2002
MAP2K1	K57N	Kinoshita-Kikuta 2018
NPM1	W288C	Falini, 2020
PIK3R1	E365K	Rudd, 2011
ERCC6	E1119L	The ICGC/TCGA Pan-Cancer Analysis of Whole Genomes Consortium, 2020
CHEK2	P536L	The ICGC/TCGA Pan-Cancer Analysis of Whole Genomes Consortium, 2020

Supplementary Table 4. Gold standard COMF mutations

Gene	Gene Name	Enzyme Activity	Most frequent Hotspot	cDNA change	COSMIC Mutation ID	Genomic coordinates (GRCh38)
DAO	D-Amino Acid Oxidase	Amino acid oxidization	p.R283W	c.847C>T	COSM2154560	chr12:108899410
MICAL2	Microtubule Associated Monooxygenase, Calponin And LIM Domain	Methionine monooxygenase	p.L99F	c.297G>T	COSM4145413	chr11:12204282
SMPD3	Sphingomyelin Phosphodiesterase 3	hydrolysis of sphingomyelin	p.D638A	c.1913A>C	COSM4261538	chr16:68361261
SMPD3	Sphingomyelin Phosphodiesterase 3	hydrolysis of sphingomyelin	p.H639P	c.1916A>C	COSM1162286	chr16:68361258
OGHDL	oxoglutarate dehydrogenase like	a-ketoglutarate dehydrogenase	p.A400T	c.1198G>A	COSM6129560	chr10:49746848
TIMP3	TIMP Metallopeptidase Inhibitor 3	Complexes with metalloproteinases	p.A199P	c.595G>C	COSM1178726	chr22:32859336

Supplementary Table 5. Top candidate change of metabolic function mutations from METIS2

Characteristic	METIS	Coban-Akdemir, 2018	Shroff, 2020	Liu, 2015
Foundation Data	Catalogue Of Somatic Mutations In Cancer (COSMIC)	Atherosclerosis Risk in Communities Study (ARIC), Exome Aggregation Consortium (ExAC), Baylor-Center for Mendelian Genomics.	Protein Data Bank	Thyroid Stimulating Hormone Receptor Mutation Database II, IARC TP53
Mutation identified	Change of metabolic function	Gain of function	Gain of function	Loss of function, gain of function, switch of function, conservation of function
Computational Method	Recursive screening	Nonsense Mediated Decay Escape Intolerance Score calculator	Deep Learning	Hidden Markov model
Predictors	Recurrence, enzyme structural analysis, conversation, pathogenicity	Transcript sequence, frameshift site, premature termination codon efficiency	Protein structure	Conversation, pathogenicity
Metabolomic analysis?	Yes	No	No	No
Output	Missense mutations	Frameshifting indel	Missense mutations	Point locations in genes
Organism	Homo sapiens	Homo sapiens	Escherichia coli	Homo sapiens

Supplementary Table 6. Comparison between computational predictors of mutated enzyume function

pCMV6-Entry	Myc-DDK-tagged ORF clone of Homo sapiens Cas-Br-M (murine) ecotropic retroviral transforming sequence (CBL) NM_005188.2	None (WT)	ATGGCCGGCAACGTGAAGAAGAGCTCTGGGGCCGGGGGCGGCAGCGGCTCCGGGGGCTCGGGTTCGGGTG<br>GCCTGATTGGGCTCATGAAGGACGCCTTCCAGCCGCACCACCACCACCACCACCTCAGCCCCCACCC<br>GCCGGGGACGGTGGACAAGAAGATGGTGGAGAAGTGCTGGAAGCTCATGGACAAGGTGGTGCGGTTGTGT<br>CAGAACCCAAAGCTGGCGCTAAAGAATAGCCCACCTTATATCTTAGACCTGCTACCAGATACCTACCAGC<br>ATCTCCGTACTATCTTGTCAAGATATGAGGGGAAGATGGAGACACTTGGAGAAAATGAGTATTTTAGGGT<br>GTTTATGGAGAATTTGATGAAGAAAACTAAGCAAACCATAAGCCTCTTCAAGGAGGGAAAAGAAAGAATG<br>TATGAGGAGAATTCTCAGCCTAGGCGAAACCTAACCAAACTGTCCCTCATCTTCAGCCACATGCTGGCAG<br>AACTAAAAGGAATCTTTCCAAGTGGACTCTTTCAGGGAGACACATTTCGGATTACTAAAGCAGATGCTGC<br>GGAATTTTGGAGAAAAGCTTTTGGGGAAAAGACAATAGTCCCTTGGAAGAGCTTTCGACAGGCTCTACAT<br>GAAGTGCATCCCATCAGTTCTGGGCTGGAGGCCATGGCTCTGAAATCCACTATTGATCTGACCTGCAATG<br>ATTATATTTCGGTTTTTGAATTTGACATCTTTACCCGACTCTTTCAGCCCTGGTCCTCTTTGCTCAGGAA<br>TTGGAACAGCCTTGCTGTAACTCATCCTGGCTACATGGCTTTTTTGACGTATGACGAAGTGAAAGCTCGG<br>CTCCAGAAATTCATTCACAAACCTGGCAGTTATATCTTCCGGCTGAGCTGTACTCGTCTGGGTCAGTGGG<br>CTATTGGGTATGTTACTGCTGATGGGAACATTCTCCAGACAATCCCTCACAATAAACCTCTCTTCCAAGC<br>ACTGATTGATGGCTTCAGGGAAGGCTTCTATTTGTTTCCTGATGGACGAAATCAGAATCCTGATCTGACT<br>GGCTTATGTGAACCAACTCCCCAAGACCATATCAAAGTGACCCAGGAACAATATGAATTATACTGTGAGA<br>TGGGCTCCACATTCCAACTATGTAAAATATGTGCTGAAAATGATAAGGATGTAAAGATTGAGCCCTGTGG<br>ACACCTCATGTGCACATCCTGTCTTACATCCTGGCAGGAATCAGAAGGTCAGGGCTGTCCTTTCTGCCGA<br>TGTGAAATTAAAGGTACTGAACCCATCGTGGTAGATCCGTTTGATCCTAGAGGGAGTGGCAGCCTGTTGA<br>GGCAAGGAGCAGAGGGAGCTCCCTCCCCAAATTATGATGATGATGATGATGAACGAGCTGATGATACTCT<br>CTTCATGATGAAGGAATTGGCTGGTGCCAAGGTGGAACGGCCGCCTTCTCCATTCTCCATGGCCCCACAA<br>GCTTCCCTTCCCCCGGTGCCACCACGACTTGACCTTCTGCCGCAGCGAGTATGTGTTCCCTCAAGTGCTT<br>CTGCTCTTGGAACTGCTTCTAAGGCTGCTTCTGGCTCCCTTCATAAAGACAAACCATTGCCAGTACCTCC<br>CACACTTCGAGATCTTCCACCACCACCGCCTCCAGACCGGCCATATTCTGTTGGAGCAGAATCCCGACCT<br>CAAAGACGCCCCTTGCCTTGTACACCAGGCGACTGTCCCTCCAGAGACAAACTGCCCCCTGTCCCCTCTA<br>GCCGCCTTGGAGACTCATGGCTGCCCCGGCCAATCCCCAAAGTACCAGTATCTGCCCCAAGTTCCAGTGA<br>TCCCTGGACAGGAAGAGAATTAACCAACCGGCACTCACTTCCATTTTCATTGCCCTCACAAATGGAGCCC<br>AGACCAGATGTGCCTAGGCTCGGAAGCACGTTCAGTCTGGATACCTCCATGAGTATGAATAGCAGCCCAT<br>TAGTAGGTCCAGAGTGTGACCACCCCAAAATCAAACCTTCCTCATCTGCCAATGCCATTTATTCTCTGGC<br>TGCCAGACCTCTTCCTGTGCCAAAACTGCCACCTGGGGAGCAATGTGAGGGTGAAGAGGACACAGAGTAC<br>ATGACTCCCTCTTCCAGGCCTCTACGGCCTTTGGATACATCCCAGAGTTCACGAGCATGTGATTGCGACC<br>AGCAGATTGATAGCTGTACGTATGAAGCAATGTATAATATTCAGTCCCAGGCGCCATCTATCACCGAGAG<br>CAGCACCTTTGGTGAAGGGAATTTGGCCGCAGCCCATGCCAACACTGGTCCCGAGGAGTCAGAAAATGAG<br>GATGATGGGTATGATGTCCCAAAGCCACCTGTGCCGGCCGTGCTGGCCCGCCGAACTCTCTCAGATATCT<br>CTAATGCCAGCTCCTCCTTTGGCTGGTTGTCTCTGGATGGTGATCCTACAACAAATGTCACTGAAGGTTC<br>CCAAGTTCCCGAGAGGCCTCCAAAACCATTCCCGCGGAGAATCAACTCTGAACGGAAAGCTGGCAGCTGT<br>CAGCAAGGTAGTGGTCCTGCCGCCTCTGCTGCCACCGCCTCACCTCAGCTCTCCAGTGAGATCGAGAACC

			TCATGAGTCAGGGGTACTCCTACCAGGACATCCAGAAAGCTTTGGTCATTGCCCAGAACAACATCGAGAT GGCCAAAAACATCCTCCGGGAATTTGTTTCCATTTCTTCTCCTGCCCATGTAGCTACCTAG

pCMV6-Entry	Myc-DDK-tagged ORF clone of Homo sapiens Cas-Br-M (murine) ecotropic retroviral transforming sequence (CBL) NM_005188.2	Y371H	ATGGCCGGCAACGTGAAGAAGAGCTCTGGGGCCGGGGGCGGCAGCGGCTCCGGGGGCTCGGGTTCGGGTG<br>GCCTGATTGGGCTCATGAAGGACGCCTTCCAGCCGCACCACCACCACCACCACCTCAGCCCCCACCC<br>GCCGGGGACGGTGGACAAGAAGATGGTGGAGAAGTGCTGGAAGCTCATGGACAAGGTGGTGCGGTTGTGT<br>CAGAACCCAAAGCTGGCGCTAAAGAATAGCCCACCTTATATCTTAGACCTGCTACCAGATACCTACCAGC<br>ATCTCCGTACTATCTTGTCAAGATATGAGGGGAAGATGGAGACACTTGGAGAAAATGAGTATTTTAGGGT<br>GTTTATGGAGAATTTGATGAAGAAAACTAAGCAAACCATAAGCCTCTTCAAGGAGGGAAAAGAAAGAATG<br>TATGAGGAGAATTCTCAGCCTAGGCGAAACCTAACCAAACTGTCCCTCATCTTCAGCCACATGCTGGCAG<br>AACTAAAAGGAATCTTTCCAAGTGGACTCTTTCAGGGAGACACATTTCGGATTACTAAAGCAGATGCTGC<br>GGAATTTTGGAGAAAAGCTTTTGGGGAAAAGACAATAGTCCCTTGGAAGAGCTTTCGACAGGCTCTACAT<br>GAAGTGCATCCCATCAGTTCTGGGCTGGAGGCCATGGCTCTGAAATCCACTATTGATCTGACCTGCAATG<br>ATTATATTTCGGTTTTTGAATTTGACATCTTTACCCGACTCTTTCAGCCCTGGTCCTCTTTGCTCAGGAA<br>TTGGAACAGCCTTGCTGTAACTCATCCTGGCTACATGGCTTTTTTGACGTATGACGAAGTGAAAGCTCGG<br>CTCCAGAAATTCATTCACAAACCTGGCAGTTATATCTTCCGGCTGAGCTGTACTCGTCTGGGTCAGTGGG<br>CTATTGGGTATGTTACTGCTGATGGGAACATTCTCCAGACAATCCCTCACAATAAACCTCTCTTCCAAGC<br>ACTGATTGATGGCTTCAGGGAAGGCTTCTATTTGTTTCCTGATGGACGAAATCAGAATCCTGATCTGACT<br>GGCTTATGTGAACCAACTCCCCAAGACCATATCAAAGTGACCCAGGAACAATATGAATTACATTGTGAGA<br>TGGGCTCCACATTCCAACTATGTAAAATATGTGCTGAAAATGATAAGGATGTAAAGATTGAGCCCTGTGG<br>ACACCTCATGTGCACATCCTGTCTTACATCCTGGCAGGAATCAGAAGGTCAGGGCTGTCCTTTCTGCCGA<br>TGTGAAATTAAAGGTACTGAACCCATCGTGGTAGATCCGTTTGATCCTAGAGGGAGTGGCAGCCTGTTGA<br>GGCAAGGAGCAGAGGGAGCTCCCTCCCCAAATTATGATGATGATGATGATGAACGAGCTGATGATACTCT<br>CTTCATGATGAAGGAATTGGCTGGTGCCAAGGTGGAACGGCCGCCTTCTCCATTCTCCATGGCCCCACAA<br>GCTTCCCTTCCCCCGGTGCCACCACGACTTGACCTTCTGCCGCAGCGAGTATGTGTTCCCTCAAGTGCTT<br>CTGCTCTTGGAACTGCTTCTAAGGCTGCTTCTGGCTCCCTTCATAAAGACAAACCATTGCCAGTACCTCC<br>CACACTTCGAGATCTTCCACCACCACCGCCTCCAGACCGGCCATATTCTGTTGGAGCAGAATCCCGACCT<br>CAAAGACGCCCCTTGCCTTGTACACCAGGCGACTGTCCCTCCAGAGACAAACTGCCCCCTGTCCCCTCTA<br>GCCGCCTTGGAGACTCATGGCTGCCCCGGCCAATCCCCAAAGTACCAGTATCTGCCCCAAGTTCCAGTGA<br>TCCCTGGACAGGAAGAGAATTAACCAACCGGCACTCACTTCCATTTTCATTGCCCTCACAAATGGAGCCC<br>AGACCAGATGTGCCTAGGCTCGGAAGCACGTTCAGTCTGGATACCTCCATGAGTATGAATAGCAGCCCAT<br>TAGTAGGTCCAGAGTGTGACCACCCCAAAATCAAACCTTCCTCATCTGCCAATGCCATTTATTCTCTGGC<br>TGCCAGACCTCTTCCTGTGCCAAAACTGCCACCTGGGGAGCAATGTGAGGGTGAAGAGGACACAGAGTAC<br>ATGACTCCCTCTTCCAGGCCTCTACGGCCTTTGGATACATCCCAGAGTTCACGAGCATGTGATTGCGACC<br>AGCAGATTGATAGCTGTACGTATGAAGCAATGTATAATATTCAGTCCCAGGCGCCATCTATCACCGAGAG<br>CAGCACCTTTGGTGAAGGGAATTTGGCCGCAGCCCATGCCAACACTGGTCCCGAGGAGTCAGAAAATGAG<br>GATGATGGGTATGATGTCCCAAAGCCACCTGTGCCGGCCGTGCTGGCCCGCCGAACTCTCTCAGATATCT<br>CTAATGCCAGCTCCTCCTTTGGCTGGTTGTCTCTGGATGGTGATCCTACAACAAATGTCACTGAAGGTTC<br>CCAAGTTCCCGAGAGGCCTCCAAAACCATTCCCGCGGAGAATCAACTCTGAACGGAAAGCTGGCAGCTGT<br>CAGCAAGGTAGTGGTCCTGCCGCCTCTGCTGCCACCGCCTCACCTCAGCTCTCCAGTGAGATCGAGAACC<br>TCATGAGTCAGGGGTACTCCTACCAGGACATCCAGAAAGCTTTGGTCATTGCCCAGAACAACATCGAGAT<br>GGCCAAAAACATCCTCCGGGAATTTGTTTCCATTTCTTCTCCTGCCCATGTAGCTACCTAG

pCMV6-Entry	Myc-DDK-tagged ORF clone of Homo sapiens Williams-Beuren syndrome chromosome region 17 (WBSCR17) NM_022479.1	None (WT)	ATGGCTTCACTGAGAAGAGTCAAAGTGCTGTTGGTGTTGAACTTGATCGCGGTAGCCGGCTTCGTGCTCT<br>TCCTGGCCAAGTGCCGGCCCATCGCGGTGCGCAGCGGAGACGCCTTCCACGAGATCCGGCCGCGCGCCGA<br>GGTGGCCAACCTCAGCGCGCACAGCGCCAGCCCCATCCAGGATGCGGTCCTGAAGCGCCTGTCGCTGCTG<br>GAGGACATCGTGTACCGGCAGCTGAATGGCTTATCCAAATCCCTTGGGCTCATTGAAGGTTATGGTGGGC<br>GGGGTAAAGGGGGCCTTCCGGCTACTCTTTCCCCGGCTGAAGAAGAAAAGGCTAAGGGACCCCATGAGAA<br>GTATGGCTACAATTCATACCTCAGTGAAAAAATTTCACTGGACCGTTCCATTCCGGATTATCGTCCCACC<br>AAGTGTAAGGAGCTCAAGTACTCCAAGGACCTGCCCCAGATATCCATCATATTCATCTTCGTGAACGAGG<br>CCCTGTCGGTGATCCTGCGGTCCGTGCACAGTGCCGTCAATCACACGCCCACACACCTGCTGAAGGAAAT<br>CATTCTGGTGGATGACAACAGCGACGAAGAGGAGCTGAAGGTCCCCCTAGAGGAGTATGTCCACAAACGC<br>TACCCCGGGCTGGTGAAGGTGGTAAGAAATCAGAAGAGGGAAGGCCTGATCCGCGCTCGCATTGAGGGCT<br>GGAAGGTGGCTACCGGGCAGGTCACTGGCTTCTTTGATGCCCACGTGGAATTCACCGCTGGCTGGGCTGA<br>GCCGGTTCTATCCCGCATCCAGGAAAACCGGAAGCGTGTGATCCTCCCCTCCATTGACAACATCAAACAG<br>GACAACTTTGAGGTGCAGCGGTACGAGAACTCGGCCCACGGGTACAGCTGGGAGCTGTGGTGCATGTACA<br>TCAGCCCCCCAAAAGACTGGTGGGACGCCGGAGACCCTTCTCTCCCCATCAGGACCCCAGCCATGATAGG<br>CTGCTCGTTCGTGGTCAACAGGAAGTTCTTCGGTGAAATTGGTCTTCTGGATCCTGGCATGGATGTATAC<br>GGAGGAGAAAATATTGAACTGGGAATCAAGGTATGGCTCTGTGGGGGCAGCATGGAGGTCCTTCCTTGCT<br>CACGGGTGGCCCACATTGAGCGGAAGAAGAAGCCATATAATAGCAACATTGGCTTCTACACCAAGAGGAA<br>TGCTCTTCGCGTTGCTGAGGTCTGGATGGACGATTACAAGTCTCATGTGTACATAGCGTGGAACCTGCCG<br>CTGGAGAATCCGGGAATTGACATCGGTGATGTCTCCGAAAGAAGAGCATTAAGGAAAAGTTTAAAGTGTA<br>AGAATTTCCAGTGGTACCTGGACCATGTTTACCCAGAAATGAGAAGATACAATAATACCGTTGCTTACGG<br>GGAGCTTCGCAACAACAAGGCAAAAGACGTCTGCTTGGACCAGGGGCCGCTGGAGAACCACACAGCAATA<br>TTGTATCCGTGCCATGGCTGGGGACCACAGCTTGCCCGCTACACCAAGGAAGGCTTCCTGCACTTGGGTG<br>CCCTGGGGACCACCACACTCCTCCCTGACACCCGCTGCCTGGTGGACAACTCCAAGAGTCGGCTGCCCCA<br>GCTCCTGGACTGCGACAAGGTCAAGAGCAGCCTGTACAAGCGCTGGAACTTCATCCAGAATGGAGCCATC<br>ATGAACAAGGGCACGGGACGCTGCCTGGAGGTGGAGAACCGGGGCCTGGCTGGCATCGACCTCATCCTCC<br>GCAGCTGCACAGGTCAGAGGTGGACCATTAAGAACTCCATCAAGTAG
pCMV6-Entry	Myc-DDK-tagged ORF clone of Homo sapiens Williams-Beuren syndrome chromosome region 17 (WBSCR17) NM_022479.1	R228C	ATGGCTTCACTGAGAAGAGTCAAAGTGCTGTTGGTGTTGAACTTGATCGCGGTAGCCGGCTTCGTGCTCT<br>TCCTGGCCAAGTGCCGGCCCATCGCGGTGCGCAGCGGAGACGCCTTCCACGAGATCCGGCCGCGCGCCGA<br>GGTGGCCAACCTCAGCGCGCACAGCGCCAGCCCCATCCAGGATGCGGTCCTGAAGCGCCTGTCGCTGCTG<br>GAGGACATCGTGTACCGGCAGCTGAATGGCTTATCCAAATCCCTTGGGCTCATTGAAGGTTATGGTGGGC<br>GGGGTAAAGGGGGCCTTCCGGCTACTCTTTCCCCGGCTGAAGAAGAAAAGGCTAAGGGACCCCATGAGAA<br>GTATGGCTACAATTCATACCTCAGTGAAAAAATTTCACTGGACCGTTCCATTCCGGATTATCGTCCCACC<br>AAGTGTAAGGAGCTCAAGTACTCCAAGGACCTGCCCCAGATATCCATCATATTCATCTTCGTGAACGAGG<br>CCCTGTCGGTGATCCTGCGGTCCGTGCACAGTGCCGTCAATCACACGCCCACACACCTGCTGAAGGAAAT<br>CATTCTGGTGGATGACAACAGCGACGAAGAGGAGCTGAAGGTCCCCCTAGAGGAGTATGTCCACAAACGC<br>TACCCCGGGCTGGTGAAGGTGGTAAGAAATCAGAAGAGGGAAGGCCTGATCTGTGCTCGCATTGAGGGCT<br>GGAAGGTGGCTACCGGGCAGGTCACTGGCTTCTTTGATGCCCACGTGGAATTCACCGCTGGCTGGGCTGA<br>GCCGGTTCTATCCCGCATCCAGGAAAACCGGAAGCGTGTGATCCTCCCCTCCATTGACAACATCAAACAG<br>GACAACTTTGAGGTGCAGCGGTACGAGAACTCGGCCCACGGGTACAGCTGGGAGCTGTGGTGCATGTACA

			TCAGCCCCCCAAAAGACTGGTGGGACGCCGGAGACCCTTCTCTCCCCATCAGGACCCCAGCCATGATAGG<br>CTGCTCGTTCGTGGTCAACAGGAAGTTCTTCGGTGAAATTGGTCTTCTGGATCCTGGCATGGATGTATAC<br>GGAGGAGAAAATATTGAACTGGGAATCAAGGTATGGCTCTGTGGGGGCAGCATGGAGGTCCTTCCTTGCT<br>CACGGGTGGCCCACATTGAGCGGAAGAAGAAGCCATATAATAGCAACATTGGCTTCTACACCAAGAGGAA<br>TGCTCTTCGCGTTGCTGAGGTCTGGATGGACGATTACAAGTCTCATGTGTACATAGCGTGGAACCTGCCG<br>CTGGAGAATCCGGGAATTGACATCGGTGATGTCTCCGAAAGAAGAGCATTAAGGAAAAGTTTAAAGTGTA<br>AGAATTTCCAGTGGTACCTGGACCATGTTTACCCAGAAATGAGAAGATACAATAATACCGTTGCTTACGG<br>GGAGCTTCGCAACAACAAGGCAAAAGACGTCTGCTTGGACCAGGGGCCGCTGGAGAACCACACAGCAATA<br>TTGTATCCGTGCCATGGCTGGGGACCACAGCTTGCCCGCTACACCAAGGAAGGCTTCCTGCACTTGGGTG<br>CCCTGGGGACCACCACACTCCTCCCTGACACCCGCTGCCTGGTGGACAACTCCAAGAGTCGGCTGCCCCA<br>GCTCCTGGACTGCGACAAGGTCAAGAGCAGCCTGTACAAGCGCTGGAACTTCATCCAGAATGGAGCCATC<br>ATGAACAAGGGCACGGGACGCTGCCTGGAGGTGGAGAACCGGGGCCTGGCTGGCATCGACCTCATCCTCC<br>GCAGCTGCACAGGTCAGAGGTGGACCATTAAGAACTCCATCAAGTAG
pCMV6-Entry	Myc-DDK-tagged ORF clone of Homo sapiens solute carrier family 17 (anion/sugar transporter), member 5 (SLC17A5) NM_012434.3	None (WT)	ATGAGGTCTCCGGTTCGAGACCTGGCCCGGAACGATGGCGAGGAGAGCACGGACCGCACGCCTCTTCTAC<br>CGGGCGCCCCACGGGCCGAAGCCGCTCCAGTGTGCTGCTCTGCTCGTTACAACTTAGCAATTTTGGCCTT<br>TTTTGGTTTCTTCATTGTGTATGCATTACGTGTGAATCTGAGTGTTGCGTTAGTGGATATGGTAGATTCA<br>AATACAACTTTAGAAGATAATAGAACTTCCAAGGCGTGTCCAGAGCATTCTGCTCCCATAAAAGTTCATC<br>ATAATCAAACGGGTAAGAAGTACCAATGGGATGCAGAAACTCAAGGATGGATTCTCGGTTCCTTTTTTTA<br>TGGCTACATCATCACACAGATTCCTGGAGGATATGTTGCCAGCAAAATAGGGGGGAAAATGCTGCTAGGA<br>TTTGGGATCCTTGGCACTGCTGTCCTCACCCTGTTCACTCCCATTGCTGCAGATTTAGGAGTTGGACCAC<br>TCATTGTACTCAGAGCACTAGAAGGACTAGGAGAGGGTGTTACATTTCCAGCCATGCATGCCATGTGGTC<br>TTCTTGGGCTCCCCCTCTTGAAAGAAGCAAACTTCTTAGCATTTCATATGCAGGAGCACAGCTTGGGACA<br>GTAATTTCTCTTCCTCTTTCTGGAATAATTTGCTACTATATGAATTGGACTTATGTCTTCTACTTTTTTG<br>GTACTATTGGAATATTTTGGTTTCTTTTGTGGATCTGGTTAGTTAGTGACACACCACAAAAACACAAGAG<br>AATTTCCCATTATGAAAAGGAATACATTCTTTCATCATTAAGAAATCAGCTTTCTTCACAGAAGTCAGTG<br>CCGTGGGTACCCATTTTAAAATCCCTGCCACTTTGGGCTATCGTAGTTGCACACTTTTCTTACAACTGGA<br>CTTTTTATACTTTATTGACATTATTGCCTACTTATATGAAGGAGATCCTAAGGTTCAATGTTCAAGAGAA<br>TGGGTTTTTATCTTCATTGCCTTATTTAGGCTCTTGGTTATGTATGATCCTGTCTGGTCAAGCTGCTGAC<br>AATTTAAGGGCAAAATGGAATTTTTCAACTTTATGTGTTCGCAGAATTTTTAGCCTTATAGGAATGATTG<br>GACCTGCAGTATTCCTGGTAGCTGCTGGCTTCATTGGCTGTGATTATTCTTTGGCCGTTGCTTTCCTAAC<br>TATATCAACAACACTGGGAGGCTTTTGCTCTTCTGGATTTAGCATCAACCATCTGGATATTGCTCCTTCG<br>TATGCTGGTATCCTCCTGGGCATCACAAATACATTTGCCACTATTCCAGGAATGGTTGGGCCCGTCATTG<br>CTAAAAGTCTGACCCCTGATAACACTGTTGGAGAATGGCAAACCGTGTTCTATATTGCTGCTGCTATTAA<br>TGTTTTTGGTGCCATTTTCTTTACACTATTCGCCAAAGGTGAAGTACAAAACTGGGCTCTCAATGATCAC<br>CATGGACACAGACACTGA
pCMV6-Entry	Myc-DDK-tagged ORF clone of Homo sapiens solute carrier family 17	R364C	ATGAGGTCTCCGGTTCGAGACCTGGCCCGGAACGATGGCGAGGAGAGCACGGACCGCACGCCTCTTCTAC<br>CGGGCGCCCCACGGGCCGAAGCCGCTCCAGTGTGCTGCTCTGCTCGTTACAACTTAGCAATTTTGGCCTT<br>TTTTGGTTTCTTCATTGTGTATGCATTACGTGTGAATCTGAGTGTTGCGTTAGTGGATATGGTAGATTCA

	(anion/sugar transporter), member 5 (SLC17A5) NM_012434.3		AATACAACTTTAGAAGATAATAGAACTTCCAAGGCGTGTCCAGAGCATTCTGCTCCCATAAAAGTTCATC<br>ATAATCAAACGGGTAAGAAGTACCAATGGGATGCAGAAACTCAAGGATGGATTCTCGGTTCCTTTTTTA<br>TGGCTACATCATCACACAGATTCCTGGAGGATATGTTGCCAGCAAAATAGGGGGGAAAATGCTGCTAGGA<br>TTTGGGATCCTTGGCACTGCTGTCCTCACCCTGTTCACTCCCATTGCTGCAGATTTAGGAGTTGGACCAC<br>TCATTGTACTCAGAGCACTAGAAGGACTAGGAGAGGGTGTTACATTTCCAGCCATGCATGCCATGTGGTC<br>TTCTTGGGCTCCCCCTCTTGAAAGAAGCAAACTTCTTAGCATTTCATATGCAGGAGCACAGCTTGGGACA<br>GTAATTTCTCTTCCTCTTTCTGGAATAATTTGCTACTATATGAATTGGACTTATGTCTTCTACTTTTTTG<br>GTACTATTGGAATATTTTGGTTTCTTTTGTGGATCTGGTTAGTTAGTGACACACCACAAAAACACAAGAG<br>AATTTCCCATTATGAAAAGGAATACATTCTTTCATCATTAAGAAATCAGCTTTCTTCACAGAAGTCAGTG<br>CCGTGGGTACCCATTTTAAAATCCCTGCCACTTTGGGCTATCGTAGTTGCACACTTTTCTTACAACTGGA<br>CTTTTTATACTTTATTGACATTATTGCCTACTTATATGAAGGAGATCCTAAGGTTCAATGTTCAAGAGAA<br>TGGGTTTTTATCTTCATTGCCTTATTTAGGCTCTTGGTTATGTATGATCCTGTCTGGTCAAGCTGCTGAC<br>AATTTAAGGGCAAAATGGAATTTTTCAACTTTATGTGTTTGTAGAATTTTTAGCCTTATAGGAATGATTG<br>GACCTGCAGTATTCCTGGTAGCTGCTGGCTTCATTGGCTGTGATTATTCTTTGGCCGTTGCTTTCCTAAC<br>TATATCAACAACACTGGGAGGCTTTTGCTCTTCTGGATTTAGCATCAACCATCTGGATATTGCTCCTTCG<br>TATGCTGGTATCCTCCTGGGCATCACAAATACATTTGCCACTATTCCAGGAATGGTTGGGCCCGTCATTG<br>CTAAAAGTCTGACCCCTGATAACACTGTTGGAGAATGGCAAACCGTGTTCTATATTGCTGCTGCTATTAA<br>TGTTTTTGGTGCCATTTTCTTTACACTATTCGCCAAAGGTGAAGTACAAAACTGGGCTCTCAATGATCAC<br>CATGGACACAGACACTGA
pCMV6-Entry	Myc-DDK-tagged ORF clone of Homo sapiens oxoglutarate dehydrogenase like (OGDHL), nuclear gene encoding mitochondrial protein, ORF from transcript variant 3, NM_001143999.1	WT	ATGTTCATCAACGATGTGGAGCAGTGCCAGTGGATCCGGCAGAAGTTTGAGACCCCTGGT<br>GTGATGCAGTTCTCCAGCGAGGAGAAGCGGACCCTGCTGGCCCGGCTAGTGCGCTCCATG<br>AGGTTTGAAGACTTCCTGGCCCGGAAATGGTCCTCAGAGAAGCGGTTTGGCCTGGAGGGC<br>TGTGAAGTGATGATTCCTGCCCTCAAGACCATCATCGACAAATCCAGCGAGATGGGGATT<br>GAGAATGTCATCTTGGGGATGCCACACAGGGGAAGGCTGAACGTGCTGGCCAACGTGATC<br>CGCAAGGACCTGGAGCAGATCTTCTGCCAGTTTGACCCCAAGCTGGAGGCGGCGGACGAG<br>GGCTCCGGGGATGTCAAGTACCACCTGGGCATGTACCATGAGAGGATCAACCGCGTCACC<br>AACCGGAACATCACTCTGTCGCTGGTTGCCAACCCCTCCCACCTGGAGGCAGTGGACCCT<br>GTGGTGCAGGGGAAGACAAAGGCAGAGCAGTTCTACCGTGGAGATGCCCAGGGCAAGAAG<br>GTCATGTCCATCCTGGTTCATGGGGACGCCGCCTTTGCTGGCCAGGGCGTGGTATATGAG<br>ACCTTCCACCTGAGCGACCTGCCCTCCTACACGACCAATGGTACCGTGCACGTCGTCGTC<br>AACAACCAGATTGGATTCACCACAGACCCCCGAATGGCCCGCTCCTCACCATACCCGACC<br>GACGTGGCCCGGGTGGTCAATGCGCCTATCTTCCATGTGAATGCCGATGACCCAGAGGCT<br>GTGATATATGTGTGCAGTGTGGCAGCCGAATGGAGAAACACTTTCAACAAAGATGTTGTC<br>GTGGACCTGGTCTGTTACCGCCGGCGTGGCCACAATGAGATGGACGAGCCCATGTTCACC<br>CAGCCGCTCATGTACAAGCAGATCCACAGACAGGTGCCTGTGCTGAAGAAGTACGCAGAC<br>AAGCTGATTGCCGAGGGCACAGTCACCCTGCAGGAGTTTGAGGAAGAAATTGCCAAATAC<br>GACCGGATCTGTGAGGAGGCTTATGGCAGGTCCAAGGATAAAAAGATTCTGCATATAAAG<br>CACTGGTTGGACTCCCCCTGGCCTGGCTTCTTCAACGTAGATGGGGAGCCCAAGAGCATG<br>ACATGCCCAGCCACGGGGATCCCTGAGGACATGCTCACCCACATCGGCAGTGTGGCCAGC

			TCTGTGCCCCTGGAGGACTTTAAGATCCACACTGGCCTCTCTCGCATTCTGCGGGGCCGT<br>GCGGACATGACCAAGAACCGGACGGTGGACTGGGCGTTGGCAGAGTACATGGCCTTTGGC<br>TCCCTGCTGAAGGAAGGCATCCACGTGCGGCTCAGCGGGCAGGATGTGGAGAGGGGCACA<br>TTCAGTCACCGGCACCATGTTCTCCATGACCAGGAGGTTGACCGCAGGACGTGTGTGCCT<br>ATGAATCATCTCTGGCCTGACCAGGCCCCGTACACCGTGTGCAACAGCTCCCTCTCGGAG<br>TACGGAGTCCTGGGCTTTGAGCTGGGCTATGCCATGGCCAGCCCCAATGCCCTGGTCCTC<br>TGGGAGGCCCAGTTTGGGGACTTCCACAACACGGCCCAGTGCATCATCGACCAGTTCATC<br>AGCACCGGCCAGGCCAAGTGGGTGCGGCATAATGGCATTGTGCTGCTGCTGCCCCATGGC<br>ATGGAAGGCATGGGCCCAGAGCACTCGTCAGCGAGGCCCGAAAGGTTCCTGCAGATGAGC<br>AATGATGACTCGGATGCCTACCCTGCATTCACCAAGGACTTCGAGGTGAGCCAGCTCTAT<br>GACTGCAACTGGATCGTGGTCAACTGCTCCACACCGGCCAACTACTTCCACGTGCTGCGC<br>CGGCAGATCCTGCTGCCCTTCCGCAAGCCGCTGATTATCTTCACACCTAAATCTCTGCTG<br>AGGCACCCAGAGGCCAAGTCCAGCTTTGACCAAATGGTATCCGGGACCAGCTTCCAGCGG<br>GTGATTCCTGAAGATGGGGCCGCAGCACGGGCCCCTGAGCAGGTGCAGCGGCTCATCTTC<br>TGCACGGGAAAGGTGTACTATGACCTGGTGAAGGAGCGGAGCAGCCAGGACCTGGAGGAG<br>AAAGTGGCCATCACGCGCCTGGAGCAGATCTCTCCATTCCCCTTCGACCTGATCAAGCAG<br>GAGGCAGAGAAGTACCCAGGTGCGGAGCTGGCCTGGTGTCAGGAGGAGCACAAGAACATG<br>GGCTACTATGACTACATCAGCCCACGCTTCATGACCATCCTGAGGCGCGCACGGCCCATA<br>TGGTATGTTGGCCGGGACCCAGCGGCTGCACCAGCCACAGGAAACAGGAACACTCACCTG<br>GTGTCACTGAAGAAGTTTCTGGATACTGCCTTCAATCTCCAGGCCTTTGAGGGCAAGACA<br>TTTTAG
pCMV6-Entry	Myc-DDK-tagged ORF clone of Homo sapiens oxoglutarate dehydrogenase like (OGDHL), nuclear gene encoding mitochondrial protein, ORF from transcript variant 3, NM_001143999.1	A191T (equivalent to A400T in full-length OGDHL)	ATGTTCATCAACGATGTGGAGCAGTGCCAGTGGATCCGGCAGAAGTTTGAGACCCCTGGT<br>GTGATGCAGTTCTCCAGCGAGGAGAAGCGGACCCTGCTGGCCCGGCTAGTGCGCTCCATG<br>AGGTTTGAAGACTTCCTGGCCCGGAAATGGTCCTCAGAGAAGCGGTTTGGCCTGGAGGGC<br>TGTGAAGTGATGATTCCTGCCCTCAAGACCATCATCGACAAATCCAGCGAGATGGGGATT<br>GAGAATGTCATCTTGGGGATGCCACACAGGGGAAGGCTGAACGTGCTGGCCAACGTGATC<br>CGCAAGGACCTGGAGCAGATCTTCTGCCAGTTTGACCCCAAGCTGGAGGCGGCGGACGAG<br>GGCTCCGGGGATGTCAAGTACCACCTGGGCATGTACCATGAGAGGATCAACCGCGTCACC<br>AACCGGAACATCACTCTGTCGCTGGTTGCCAACCCCTCCCACCTGGAGGCAGTGGACCCT<br>GTGGTGCAGGGGAAGACAAAGGCAGAGCAGTTCTACCGTGGAGATGCCCAGGGCAAGAAG<br>GTCATGTCCATCCTGGTTCATGGGGACGCCACATTTGCTGGCCAGGGCGTGGTATATGAG<br>ACCTTCCACCTGAGCGACCTGCCCTCCTACACGACCAATGGTACCGTGCACGTCGTCGTC<br>AACAACCAGATTGGATTCACCACAGACCCCCGAATGGCCCGCTCCTCACCATACCCGACC<br>GACGTGGCCCGGGTGGTCAATGCGCCTATCTTCCATGTGAATGCCGATGACCCAGAGGCT<br>GTGATATATGTGTGCAGTGTGGCAGCCGAATGGAGAAACACTTTCAACAAAGATGTTGTC<br>GTGGACCTGGTCTGTTACCGCCGGCGTGGCCACAATGAGATGGACGAGCCCATGTTCACC<br>CAGCCGCTCATGTACAAGCAGATCCACAGACAGGTGCCTGTGCTGAAGAAGTACGCAGAC<br>AAGCTGATTGCCGAGGGCACAGTCACCCTGCAGGAGTTTGAGGAAGAAATTGCCAAATAC<br>GACCGGATCTGTGAGGAGGCTTATGGCAGGTCCAAGGATAAAAAGATTCTGCATATAAAG

			CACTGGTTGGACTCCCCCTGGCCTGGCTTCTTCAACGTAGATGGGGAGCCCAAGAGCATG<br>ACATGCCCAGCCACGGGGATCCCTGAGGACATGCTCACCCACATCGGCAGTGTGGCCAGC<br>TCTGTGCCCCTGGAGGACTTTAAGATCCACACTGGCCTCTCTCGCATTCTGCGGGGCCGT<br>GCGGACATGACCAAGAACCGGACGGTGGACTGGGCGTTGGCAGAGTACATGGCCTTTGGC<br>TCCCTGCTGAAGGAAGGCATCCACGTGCGGCTCAGCGGGCAGGATGTGGAGAGGGGCACA<br>TTCAGTCACCGGCACCATGTTCTCCATGACCAGGAGGTTGACCGCAGGACGTGTGTGCCT<br>ATGAATCATCTCTGGCCTGACCAGGCCCCGTACACCGTGTGCAACAGCTCCCTCTCGGAG<br>TACGGAGTCCTGGGCTTTGAGCTGGGCTATGCCATGGCCAGCCCCAATGCCCTGGTCCTC<br>TGGGAGGCCCAGTTTGGGGACTTCCACAACACGGCCCAGTGCATCATCGACCAGTTCATC<br>AGCACCGGCCAGGCCAAGTGGGTGCGGCATAATGGCATTGTGCTGCTGCTGCCCCATGGC<br>ATGGAAGGCATGGGCCCAGAGCACTCGTCAGCGAGGCCCGAAAGGTTCCTGCAGATGAGC<br>AATGATGACTCGGATGCCTACCCTGCATTCACCAAGGACTTCGAGGTGAGCCAGCTCTAT<br>GACTGCAACTGGATCGTGGTCAACTGCTCCACACCGGCCAACTACTTCCACGTGCTGCGC<br>CGGCAGATCCTGCTGCCCTTCCGCAAGCCGCTGATTATCTTCACACCTAAATCTCTGCTG<br>AGGCACCCAGAGGCCAAGTCCAGCTTTGACCAAATGGTATCCGGGACCAGCTTCCAGCGG<br>GTGATTCCTGAAGATGGGGCCGCAGCACGGGCCCCTGAGCAGGTGCAGCGGCTCATCTTC<br>TGCACGGGAAAGGTGTACTATGACCTGGTGAAGGAGCGGAGCAGCCAGGACCTGGAGGAG<br>AAAGTGGCCATCACGCGCCTGGAGCAGATCTCTCCATTCCCCTTCGACCTGATCAAGCAG<br>GAGGCAGAGAAGTACCCAGGTGCGGAGCTGGCCTGGTGTCAGGAGGAGCACAAGAACATG<br>GGCTACTATGACTACATCAGCCCACGCTTCATGACCATCCTGAGGCGCGCACGGCCCATA<br>TGGTATGTTGGCCGGGACCCAGCGGCTGCACCAGCCACAGGAAACAGGAACACTCACCTG<br>GTGTCACTGAAGAAGTTTCTGGATACTGCCTTCAATCTCCAGGCCTTTGAGGGCAAGACA<br>TTTTAG

Supplementary Table 7. cDNA sequences for plasmids used in this study

Supplementary Note

Supplementary description of METIS1 hits.

First, we identified a p.Y371H mutation in the E3 Ubiquitin ligase Cas-Br-M ectropic retroviral transforming sequence (CBL). These mutations occurred in a highly recurrent fashion (**Fig. 2a**). The residue is highly conserved in eukaryotes (**Fig. 2b**) and is located within a critical region of the CBL protein (**Fig. 2c**).

Second, we identified a recurrent p.R228C mutation in the protein-UDP acetylgalactosyltransferase named Williams-Beuren syndrome chromosome region 17 (WBSCR17, now renamed to GALNT17). Identical p.R228C mutations were found in a melanoma sample and two colorectal cancer samples (**Fig. 2d**). The residue is conserved in vertebrates (**Fig. 2e**). We examined a structure available for the mouse homolog UDP-GalNAc polypeptide alpha-N-acetylgalactosaminyltransferase-T1, and the R228 residue is located within the active site of the WBSCR17 protein (**Fig. 2f**).

Third, we identified a recurrent p.R364C mutation in the anion/sugar transporter Solute Carrier Family 17 (SLC17A5), also known as Sialin. SLC17A5 is critical for the transport of the important sugar, sialic acid, out of lysosomes, and deficiency in SLC17A5 causes Salla disease. We elected to include solute carriers and transporters along with enzymes, as we reasoned that mutations could cause useful GOFs that

could help transport desired biochemicals for metabolic engineering applications. Identical SLC17A5 p.R364C mutations were found in two melanoma samples and one colorectal cancer sample (**Fig. 2g**). The residue is conserved as distantly as in plants (**Fig. 2h**). No protein structure data was available to visualize p.R364 on SLC17A5.

Fourth we identified recurrent p.A400T mutations in oxoglutarate dehydrogenase-like (OGDHL). OGDHL likely encodes an additional isoform of a-ketoglutarate dehydrogenase, which converts a-ketoglutarate to succinyl-CoA and produces NADH for the respiratory chain[1]. The OGDHL p.A400T mutations were found in three colorectal cancers (**Fig. 2i**). An additional case contained a p.A400S mutation. p.A400 is conserved in vertebrates (**Fig. 2j**). We searched for structures of homologs of OGDHL and could only identify distant homologs, with the closest homolog from Mycobacterium smegmatis alpha-ketoglutarate decarboxylase[2]. Since enzyme structures tend to be highly conserved even across distantly-related species, we examined this structure. The analogous residue to human OGDHL A400 mapped within the active site within 10 A of the ligand (**Fig. 2k**).

Supplementary metabolic pathway analysis.

Several amino acids were increased in cells expressing CBL WT compared to CBL p.Y371H (**Fig. 4a**). 5-oxoproline, leucine, and methionine sulfoxide in the amino acid metabolism were significantly increased in the CBL WT group compared to the CBL mutant group ($P<0.05$, **Extended Data Fig. 2**). These may reflect freed amino acids released from proteasomal degradation related to the normal E3 ubiquitin ligase

functions of CBL WT [3]. No biochemicals were significantly increased in the CBL mutant group compared to the CBL-WT. Given the lack of changes induced by the CBL mutant, we hypothesized that the CBL mutations may either be loss of function or that they do not affect cellular biochemical metabolism. Consistent with these findings, it has since emerged that CBL p.Y371H mutations are loss-of-function mutations that disrupt E3 ubiquitin ligase function[4]. These results suggest that the metabolite profiling approach can be used to identify candidate mutations that are actually loss-of-function mutations.

The WBSCR17 mutant, compared to WBSCR WT, was associated with changes in lipids including carnitine metabolites (**Fig. 4b**). Two biochemicals were increased in the WBSCR17 mutant compared to WBSCR17-WT (1-palmitoyl-glycerophosphoglycerol FC=1.5 and P=0.03, and 1-methyladenosine FC=1.3 and P=0.02, **Extended Data Fig. 3a**). In contrast, multiple carnitine metabolites were increased in the WT group compared to the WBSCR17 mutant (acetylcarnitine, butyrylcarnitine, deoxycarnitine, 2-methyl-butyryl carnitine; P<0.05 for each, **Extended Data Fig. 3b**). Also, gluconate, NAD+, and cysteinylglycine were increased in the WT group (P<0.05, **Extended Data Fig. 3c**). Thus, WBSCR17 changes are relatively small (all <1.7 fold change between WT and mutant). Also, no changes exhibited a significant difference from all other samples after FDR correction (see **Fig. 3b**). We conclude that a GOF could not be identified for the WBSCR17 mutant from this metabolite profiling dataset.

The SLC17A5 mutant was associated with changes in energy and nucleoside metabolism. Five biochemicals were increased in the SLC17A5 mutant group compared to the SLC17A5 WT group (taurine, butyrylcarnitine, acetylcarnitine, deoxycarnitine, 5'-GMP; FC range 1.4-1.8, $P<0.05$ for each, **Fig. 4c**). SLC17A5 mutants possessed significantly and non-significantly elevated levels of ADP, AMP, GDP, 5'-GMP, UTP, UDP, UMP, CTP, CDP, and CMP compared to wild-type counterparts (**Extended Data Fig. 4a**). These observations may reflect increased availability for DNA and RNA synthesis and potentially result from a decrease in purine and pyrimidine degradation as suggested by lower levels of the catabolic products adenosine, xanthine, hypoxanthine, and uracil (**Extended Data Fig. 4b**). These findings could reflect cellular energy homeostasis caused by an alteration in the SLC17A5 sialic acid/proton pump function, but a direct mechanism is not clear[5].

Supplementary Statistical Justification of Recurrence

We determined that a mutation would have to occur 3+ instances to determine if an amino acid mutation was significantly recurrent. We assumed that there were 20,000 genes in the genome and that the average gene transcript length is 1950 nucleotides, or 650 codons [6,7]. Thus, there were approximately 13,000,000 codons in the human genome. There were approximately 200,000 missense mutations in COSMIC, so there was a possibility of 7.6×10^{-8} that a missense mutation was located in a codon. We used a binomial distribution function to determine the probability of X mutations occuring. We then used a battery of false discovery rate tests, including BH, Holm, and Benjamini-Yekutieli, which were calculated using R. We set $adjP<0.0001$ for all FDR tests, and 3+ occurrences were determined to be significant for a gene to be recurrent.

Here, we opted to use a strict significance criteria to limit false positives as much as possible from our proof of concept of METIS1 but this selection criterion can be loosened/tightened in future iterations of the pipeline.
