# Supplementary material for: Mining cancer genomes for change-of-metabolic-function mutations
Source: Commun Biol. 2023 Nov 10;6:1143. doi: 10.1038/s42003-023-05475-w (PMC10638295; doi:10.1038/s42003-023-05475-w)
Supplement: Supplementary file 3 — Description of additional files [file 42003_2023_5475_MOESM3_ESM.docx]

**Mining Cancer Genomes for Change-of-Metabolic-Function Mutations to Design Novel Catalysts**

**Description of Additional Files**

Kevin J. Tu, Bill H. Diplas, Joshua A. Regal, Matthew S. Waitkus, Christopher J. Pirozzi, Zachary J. Reitman

| **File Name** | **Contents** | **Description** |
| --- | --- | --- |
| Supplementary Data.xlsx | Supplementary Data 1 | Raw non-normalized ion counts from global metabolite profiling and bradford results |
| Supplementary Data.xlsx | Supplementary Data 2 | Normalized ion counts from global metabolite profiling. |
| Supplementary Data.xlsx | Supplementary Data 3 | Metabolic Pathway Analysis |
| Supplementary Code.xlsx | Supplementary Code 1 | METIS1 R code |
| Supplementary Code.xlsx | Supplementary Code 2 | METIS1 Excel steps to use as an alternative approach |
| Supplementary Code.xlsx | Supplementary Code 3 | METIS2 R code |
