# Supplementary material for: Mining cancer genomes for change-of-metabolic-function mutations
Source: Commun Biol. 2023 Nov 10;6:1143. doi: 10.1038/s42003-023-05475-w (PMC10638295; doi:10.1038/s42003-023-05475-w)
Supplement: Supplementary file 6 — Reporting-Summary [file 42003_2023_5475_MOESM6_ESM.pdf]

nature portfolio

Corresponding author(s): Zachary J. Reitman

Last updated by author(s): 10/5/2023

Reporting Summary

Nature Portfolio wishes to improve the reproducibility of the work that we publish. This form provides structure for consistency and transparency in reporting. For further information on Nature Portfolio policies, see our Editorial Policies and the Editorial Policy Checklist.

Statistics

For all statistical analyses, confirm that the following items are present in the figure legend, table legend, main text, or Methods section.

n/a	Confirmed	
☐	☒	The exact sample size (*n*) for each experimental group/condition, given as a discrete number and unit of measurement
☐	☒	A statement on whether measurements were taken from distinct samples or whether the same sample was measured repeatedly
☐	☒	The statistical test(s) used AND whether they are one- or two-sided<br>*Only common tests should be described solely by name; describe more complex techniques in the Methods section.*
☐	☒	A description of all covariates tested
☐	☒	A description of any assumptions or corrections, such as tests of normality and adjustment for multiple comparisons
☐	☒	A full description of the statistical parameters including central tendency (e.g. means) or other basic estimates (e.g. regression coefficient) AND variation (e.g. standard deviation) or associated estimates of uncertainty (e.g. confidence intervals)
☐	☒	For null hypothesis testing, the test statistic (e.g. *F*, *t*, *r*) with confidence intervals, effect sizes, degrees of freedom and *P* value noted<br>*Give P values as exact values whenever suitable.*
☒	☐	For Bayesian analysis, information on the choice of priors and Markov chain Monte Carlo settings
☒	☐	For hierarchical and complex designs, identification of the appropriate level for tests and full reporting of outcomes
☒	☐	Estimates of effect sizes (e.g. Cohen's *d*, Pearson's *r*), indicating how they were calculated

Our web collection on statistics for biologists contains articles on many of the points above.

Software and code

Policy information about availability of computer code

Data collection	COSMIC mutational datasets were downloaded from https://cancer.sanger.ac.uk/cosmic. Metabolite profiling data were collected using standard techniques that have been described in the literature (for instance https://doi.org/10.1073/pnas.1019393108).
Data analysis	Code for filtering cancer mutational datasets is provided in the Supplementary Code 1 and Supplementary Code 2 objects.

For manuscripts utilizing custom algorithms or software that are central to the research but not yet described in published literature, software must be made available to editors and reviewers. We strongly encourage code deposition in a community repository (e.g. GitHub). See the Nature Portfolio guidelines for submitting code & software for further information.

Data

Policy information about availability of data

All manuscripts must include a data availability statement. This statement should provide the following information, where applicable:

- Accession codes, unique identifiers, or web links for publicly available datasets
- A description of any restrictions on data availability
- For clinical datasets or third party data, please ensure that the statement adheres to our policy

We generated metabolite profiling datasets that are available in the supplementary tables. Outputs from filtering of publicly-available COSMIC mutation databases are also included in the Supplementary Tables.

Research involving human participants, their data, or biological material

Policy information about studies with human participants or human data. See also policy information about sex, gender (identity/presentation), and sexual orientation and race, ethnicity and racism.

Reporting on sex and gender	No human subjects were used in the study. We do not report on sex or gender.
Reporting on race, ethnicity, or other socially relevant groupings	No human subjects were used in the study. We do not report on race, ethnicity, or other socially relevant groupings.
Population characteristics	See above.
Recruitment	No human subjects were used in the study.
Ethics oversight	No human subjects were used in the study.

Note that full information on the approval of the study protocol must also be provided in the manuscript.

Field-specific reporting

Please select the one below that is the best fit for your research. If you are not sure, read the appropriate sections before making your selection.

☒ Life sciences ☐ Behavioural & social sciences ☐ Ecological, evolutionary & environmental sciences

For a reference copy of the document with all sections, see nature.com/documents/nr-reporting-summary-flat.pdf

Life sciences study design

All studies must disclose on these points even when the disclosure is negative.

Sample size	We used all available data in a cancer mutational dataset. We examined n=3 biological replicates per group for cell line data.
Data exclusions	No data were excluded from the analyses.
Replication	We replicated the cancer mutational data analysis with a larger, updated COSMIC dataset and found similar results, suggesting reproducibility.
Randomization	This is not relevant to our study since we did not have experimental groups that could be randomized.
Blinding	This is not relevant to our study since we did not have experimental groups that could be blinded

Reporting for specific materials, systems and methods

We require information from authors about some types of materials, experimental systems and methods used in many studies. Here, indicate whether each material, system or method listed is relevant to your study. If you are not sure if a list item applies to your research, read the appropriate section before selecting a response.

Materials & experimental systems

n/a	Involved in the study	
☐	☒	Antibodies
☐	☒	Eukaryotic cell lines
☒	☐	Palaeontology and archaeology
☒	☐	Animals and other organisms
☒	☐	Clinical data
☒	☐	Dual use research of concern
☒	☐	Plants

Methods

n/a	Involved in the study	
☒	☐	ChIP-seq
☒	☐	Flow cytometry
☒	☐	MRI-based neuroimaging

Antibodies

Antibodies used	DDK (FLAG) mouse monoclonal antibody, clone OTI11C3
Validation	The antibody was used on positive and negative controls with expected results.

Eukaryotic cell lines

Policy information about cell lines and Sex and Gender in Research

Cell line source(s)	The HeLa cell line was obtained from ATCC.
Authentication	None of the cell lines used were authenticated.
Mycoplasma contamination	All lines were routinely tested every 3 months at least for mycoplasma contamination and were negative.
Commonly misidentified lines (See ICLAC register)	HeLa is a commonly misidentified line. To control for cell line variability, all experiments were carried out on isogenic cells (ie, cells were split into groups at the same time and subjected to plasmid transfection and metabolomic analysis).
